# Plexin C1 modulates metabolic programming for resolution of severe inflammation

**DOI:** 10.1186/s12964-025-02518-z

**Published:** 2025-11-25

**Authors:** Andreas Körner, Michael Koeppen, Jasvir Kaur, Julia C. Fitzgerald, Sarantos Kostidis, Torsten Kaussen, Christoph Trautwein, Martin Giera, Tamam Bakchoul, Alice Bernard, Valbona Mirakaj

**Affiliations:** 1 Department of Anesthesiology and Intensive Care Medicine, Molecular Intensive Care Medicine, University Hospital Eberhard Karls University, Tübingen, Germany; 2 Department of Anesthesiology and Intensive Care Medicine, University Hospital Eberhard Karls University, Tübingen, Germany; 3 Hertie Institute for Clinical Brain Research, University Clinic Tübingen, Tübingen, Germany; 4 Center for Proteomics and Metabolomics, Leiden University Medical Center (LUMC), Leiden, The Netherlands; 5 Department of Pediatric Cardiology and Pediatric Intensive Care Medicine, Hannover Medical School, Hannover, Germany; 6 Werner Siemens Imaging Center, Department of Preclinical Imaging and Radiopharmacy, Eberhard Karls University, Tübingen, Germany; 7 Core Facility Metabolomics, Medical Faculty of Tübingen, University of Tübingen, Tübingen, Germany; 8 Transfusion Medicine, Medical Faculty of Tübingen, University Hospital of Tübingen, Tübingen, Germany; 9 Department of Anesthesiology, Intensive Care Medicine, and Pain, Translational Intensive Care Medicine, University Hospital Goethe University, Frankfurt, Germany

**Keywords:** Plexin C1, Inflammation, Leukocytes, Metabolism, Lipid mediators, Resolution, Neuronal guidance proteins, Macrophage phenotype, Regeneration

## Abstract

Persistent, unresolved inflammatory processes disrupt the complex homeostatic state and lead to severe disease, ultimately resulting in organ failure, shock, and death. The mediators that monitor and control the programmed mechanisms in tissue homeostasis and repair remain elusive. A recently discovered group of endogenous proteins, termed neuronal guidance proteins (NGPs), has gained attention as important mediators of immunoregulation and tissue homeostasis through their role in the complex interplay between metabolic reprogramming and immunity. In murine peritonitis, the deficiency of the NGP Plexin C1 (PLXC1^−/−^) led to an increase in neutrophil and Ly6C^hi^ monocyte recruitment to the injury site and a decrease in the phagocytosis rate. This was accompanied by a decrease in the endogenous biosynthesis of specialized proresolving lipid mediators (SPMs) such as Maresin-1 and Protectin DX and finally by a lengthening of the resolution interval. Peritoneal macrophages (MΦs)^PLXC1−/−^ followed specific strategies to adapt their metabolism to catabolic conditions, by reducing fatty acid oxidation and oxidative phosphorylation, increasing aerobic glycolysis and activation of the pentose phosphate pathway, and disrupting the tricarboxylic acid cycle. The decreased MΦs^PLXC1−/−^ citrate levels corresponded with a lipid mediator profile in which PGD_2_ and PGE_2_ were greatly decreased in murine PLXC1^−/−^ peritoneal exudates, indicating a lesser effect on lipid mediator class switching and ultimately the formation of SPMs. Analysis of the related signaling networks indicated mTOR and AKT2 phosphorylation pathways are critical determinants of metabolic reprogramming in activated MΦs^PLXC1−/−^. These findings highlight a novel role for PLXC1 in regulating the crosstalk between immunometabolism and inflammation resolution programs in severe inflammation.

**Supplementary Information:**

The online version contains supplementary material available at 10.1186/s12964-025-02518-z.

## Significance statement

Persistent inflammation can disrupt tissue homeostasis, leading to severe diseases like sepsis. This study identifies Plexin C1 (PLXC1), a neuronal guidance protein, as a critical regulator of inflammation resolution. In a murine peritonitis model, PLXC1 deficiency impaired MΦ metabolism, reducing the production of specialized proresolving lipid mediators (SPMs) and delaying inflammation resolution. PLXC1 also influenced key signaling pathways, metabolic reprogramming, and macrophage polarization, linking immunometabolism to tissue repair. Clinical data suggest PLXC1 may predict outcomes in critically ill children with abdominal compartment syndrome. These findings highlight PLXC1’s novel role in coordinating immune responses and inflammation resolution, offering insights into potential therapeutic strategies for managing severe inflammatory conditions.

## Main text

Severe inflammatory responses can either accomplish the resolution of inflammation or be dominated by nonresolving mechanisms that can lead to tissue destruction and loss of functional organ integrity. These pathophysiological mechanisms are most evident in syndromes such as ARDS or sepsis. Physiological mechanisms of the immune system, however, are intended to activate homeostatic mechanisms to eliminate pathogens or other harmful agents. In this context, resolution processes are highly important [1, 2]. These are characterized by spatially and temporally controlled cellular events and by the release or generation of immunoresolvents such as specialized proresolving lipid mediators (SPMs) or neuronal guidance molecules. Emerging evidence has revealed a strong coordination of immune cells with the metabolic system, which ultimately specifies cell function and fate. In this regard, monocytes and macrophages (MΦs), which play a central role in inflammation resolution processes and consequently the induction of catabasis, are of great importance [2]. In the context of an inflammatory response, metabolic reprogramming occurs to meet cellular requirements such as apoptosis, phagocytosis, proliferation, cytokine release, and the release of immuneresolvents.

A pattern for neuronal guidance proteins (NGPs) and their target receptors exists not only in axon guidance during development, but also in immunoregulation which guides leukocyte migration outside the CNS [3, 4]. Plexin C1 (PLXC1), a transmembrane receptor of secreted and membrane-bound semaphorin 7A, was originally described regarding its influence on cell adhesion and migration in a variety of immune cells [5, 6]. Subsequent work has shown that PLXC1 performs crucial nonneuronal functions in the initial phase of acute inflammation, such as in the context of acute lung and liver injury, peritonitis, or bacterial sepsis [7, 8]. The critical issue in acute inflammation is not the initiation of an inflammatory response, but the subsequent failure in adequately resolving the exacerbation of inflammatory processes and thus converting back to processes of tissue regeneration and ultimately homeostasis [1, 9, 10]. In light of these considerations coupled with the immunomodulatory effects of PLXC1 on the initiation of acute inflammation, we investigated the role of PLXC1 particularly on the mechanisms of resolution processes. In the present report, we demonstrate the specific immunomodulatory effects of PLXC1 on the resolution of acute severe inflammation. PLXC1 affects the phagocytic capacity of MΦs. MΦs^PLXC1−/−^ follow specific strategies to adapt their metabolism to catabolic conditions. The mTOR and AKT2 phosphorylation pathways were found to be critical determinants in activated MΦs^PLXC1−/−^. PLXC1 expression deficiency impairs the resolution of murine peritonitis and is clinically correlated with the outcomes in critically ill pediatric patients with abdominal compartment syndrome.

## Results

### Plexin C1 impacts MΦ phagocytic capacity

**Fig. 1:**
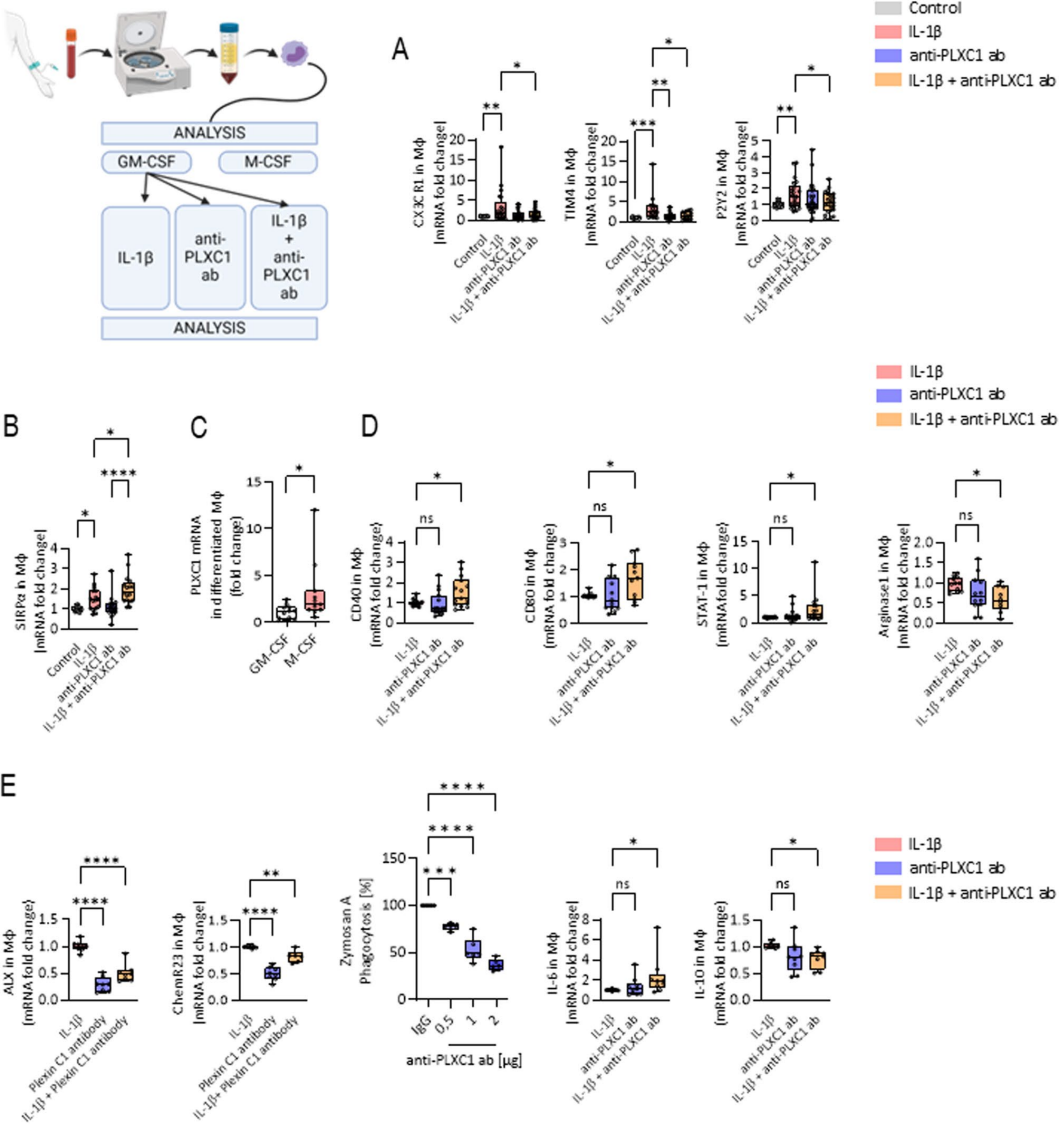
PLXC1 Modulates MΦ Polarization and Alters Proresolving Receptor Expression. Human PBMCs were stimulated with GM-CSF or M-CSF in RPMI 1640 medium supplemented with 10% fetal calf serum (FCS) for 7 days to differentiate into M1 or M2 MΦ. Subsequently, M1 MΦs were stimulated with IL-1β alone, anti-PLXC1 antibody alone, or the combination of IL-1β + anti- PLXC1 for 24 h. Transcript expression of CX3CR1, TIM4, and P2Y2, critical receptors for the “find me” signal involved in apoptotic cell clearance (**A**), and the expression of the “don’t eat me” receptor SIRPα (**B**) were analyzed. PLXC1 mRNA levels in M-CSF differentiated MΦs compared to GM-CSF differentiated MΦs were measured (**C**). In GM-CSF differentiated MΦs, the expression of M1 markers (CD40, CD80, and STAT-1) and the M2 marker arginase were evaluated under the same stimulation conditions (IL-1β, anti-PLXC1, IL-1β + anti-PLXC1) (**D**). Additionally, the expression levels of specialized pro-resolving mediators (SPM) receptors ALX and Chem23, efferocytosis rates of ZyA particles, and cytokine levels of IL-10 and IL-6 were quantified. Expression was measured by RT-PCR after RNA extraction using TRIzol reagent, cDNA synthesis with the iScript™ cDNA Synthesis Kit, and normalization to 18 S using the ΔΔCq method. The results represent 3–8 independent experiments (total *n* = 11–27) and are presented as box-and-whisker plots showing all individual data points, with whiskers indicating the minimum and maximum values; significance was determined by one-way ANOVA with Bonferroni correction (**A**, **B**, **D**, **E**) or the unpaired two-tailed Student’s t-test (**E**), *P < 0.05; **P < 0.01; ***P < 0.001, ****P < 0.0001

It is now well established that adequate clearance of dying cells is a basic prerequisite for successful resolution of inflammatory processes [11]. Early, in the so-called “transition phase” of inflammation, the process of polymorphonuclear (PMN) cell apoptosis initiates the mechanisms for resolution of inflammation. In this process, a variety of soluble signals are released, mainly to attract MΦs and activate their phagocytic capacity. To follow up on this aspect, human MΦs were stimulated with vehicle or IL-1β and/or an anti-PLXC1 antibody (Ab) and the expression of CX_3_CR_1_, TIM4 and P2Y2 - three crucial receptors of the “find me” signal - was significantly reduced following stimulation with anti-PLXC1 (Fig. 1A), whereas other receptors such as CD88, S1PR1 and G2A remained unaltered (Suppl. Figure 7A-C). This finding underscores the specificity of the signaling pathway identified in our study and suggests that Plexin C1 selectively activates distinct “find me” signaling axes. In contrast, the expression of the “don’t eat me” receptor SIRPα was strongly increased after stimulation with IL-1β and anti-PLXC1 (Fig. 1B). Thus, PLXC1 might be involved in the recognition of dying cells and might thus be involved very early in the process of resolution. Due to their preeminent plasticity, monocytes and MΦs have been shown to play an essential role in inflammation-resolution programs and in the restoration of tissue homeostasis by adapting their phenotypes and thus their functions to the pathophysiological changes [12, 13]. To investigate the possible effect of PLXC1 on MΦ phenotypic polarization, we first examined PLXC1 expression in GM-CSF and M-CSF differentiated MΦs and showed that PLXC1-mRNA and protein levels were significantly greater in M-CSF differentiated MΦs (Fig. 1C, Suppl. Figure 5C). In GM-CSF differentiated MΦs, the expression of the M1 markers CD40, CD80 and STAT-1 was markedly increased, whereas the M2 marker arginase was significantly decreased following stimulation with IL-1β and anti-PLXC1 (Fig. 1D, Suppl. Figure 5A-B). This was also reflected in the expression of the SPM receptors ALX and Chem23, the phagocytosis rate and levels of inflammatory cytokines (Fig. 1E, Suppl. Figs. 6 and 8). Thus, these findings suggest that PLXC1 shifts the polarization of MΦs toward the M2 phenotype, presumably promoting the activation of resolution processes.

### MΦs^PLXC1−/−^ follow specific strategies to adapt their metabolism to catabolic conditions

**Fig. 2:**
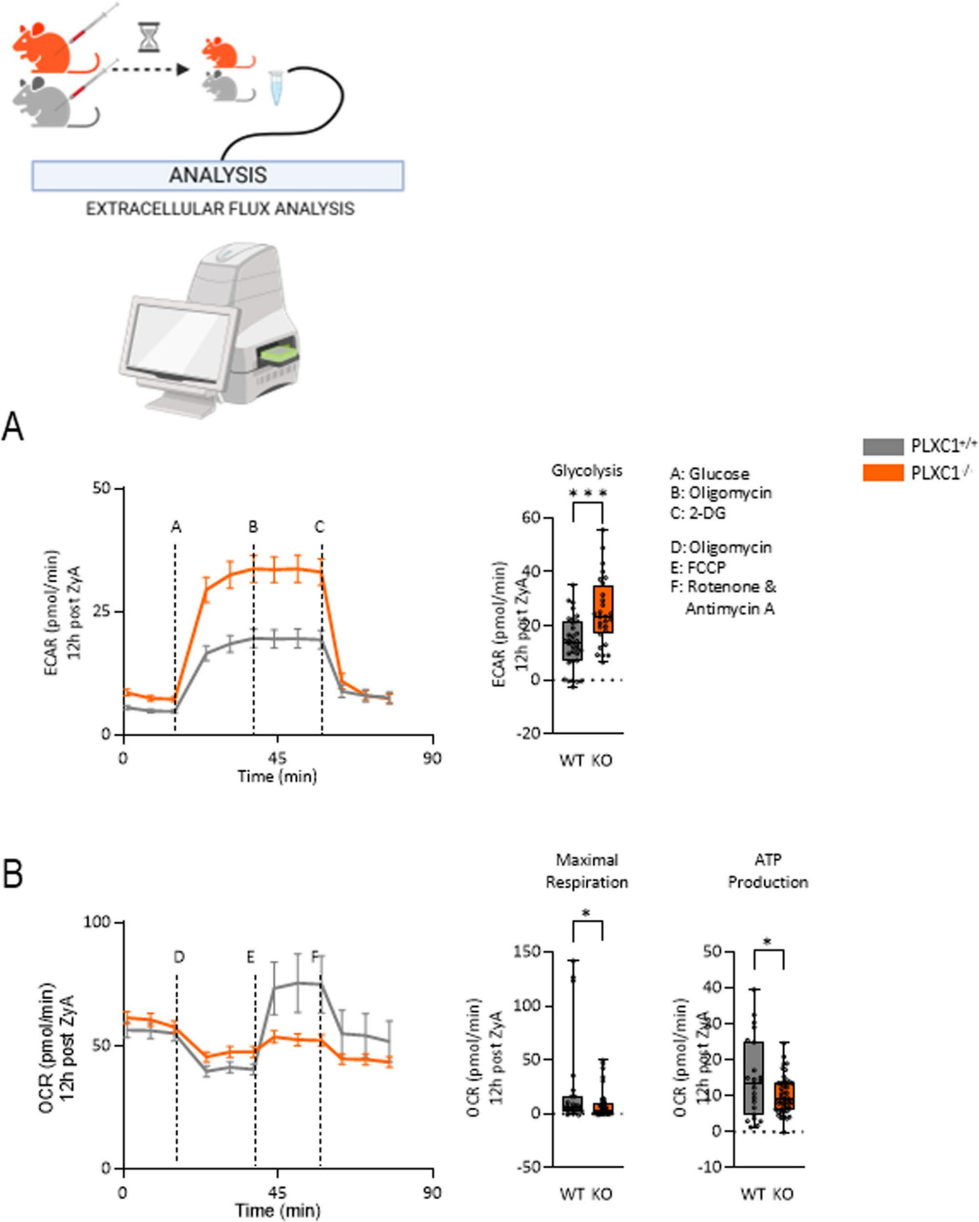
PLXC1 Deficiency Alters MΦ Glycolytic Capacity and Mitochondrial Respiration. Glycolytic capacity and mitochondrial respiration were assessed using the Seahorse Glycolysis and Mito Stress tests. Peritoneal macrophages (MΦs) from PLXC1^+/+^ and PLXC1^-/-^ mice, pretreated with ZyA for 12 h, were analyzed for extracellular acidification rates (ECARs) after sequential injections of glucose, oligomycin, and 2-DG (**A**). Oxygen consumption rates (OCRs) were determined following injections of oligomycin, FCCP, and Rotenone & Antimycin A (**B**). Samples were pooled from 3 to 4 mice per group. The results represent at least three independent experiments and are expressed as the mean ± SEM (**A**, **B** left panel) or as box-and-whisker plots (**A**, **B** right panels) showing all individual data points, with whiskers indicating the minimum and maximum values (**B**); significance was determined by the unpaired two-tailed Student’s t-test, *P < 0.05; ***P < 0.001

**Fig. 3:**
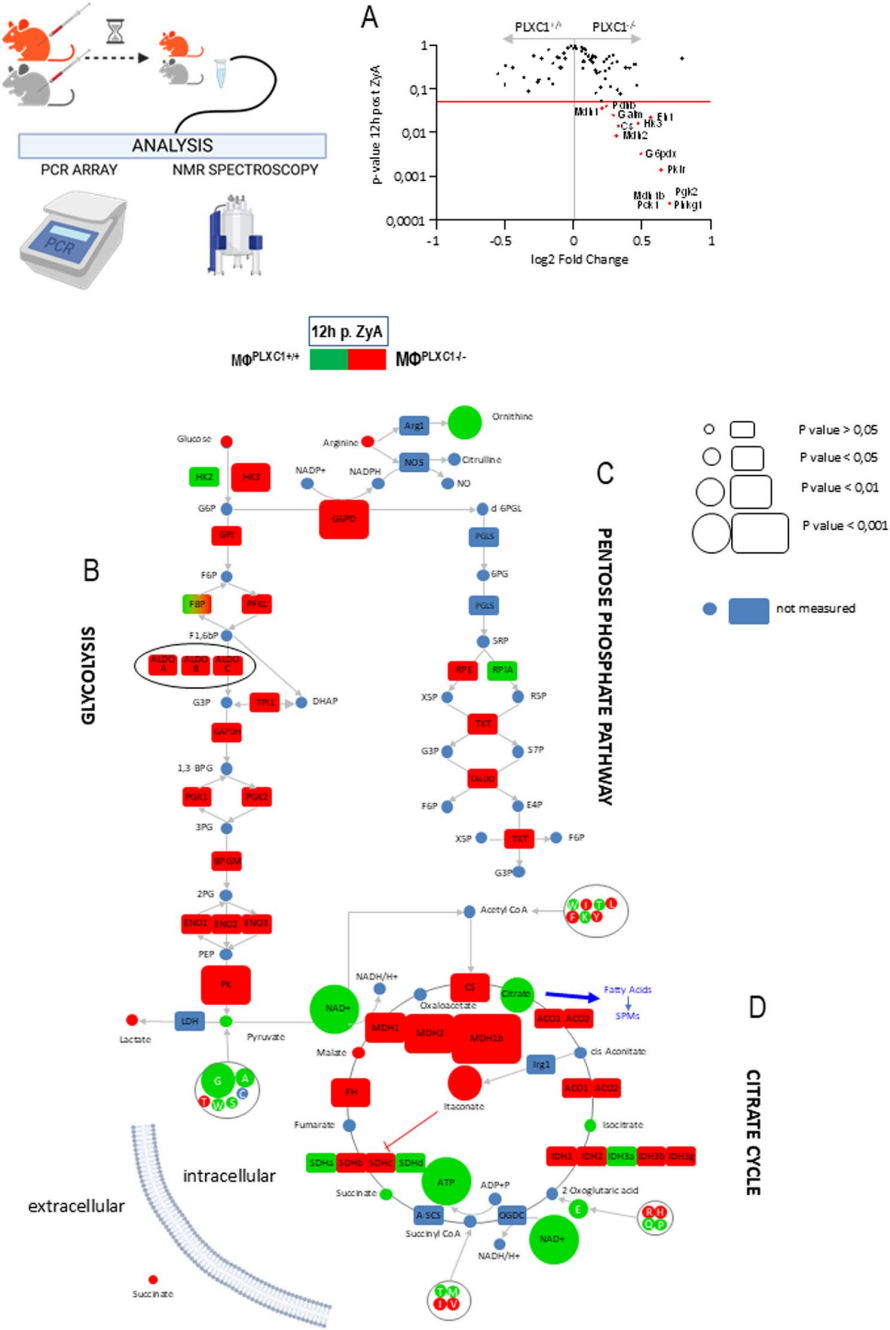
PLXC1 Deficiency Alters MΦ – specific strategies of Metabolic Adaptions. The glucose metabolism pathways in PLXC1^+/+^ MΦs and PLXC1^-/-^ MΦs were assessed via PCR-based analysis of enzymes involved in glucose metabolism (**A**). Intracellular metabolites were quantified by NMR spectroscopy to investigate glycolysis (**B**), the pentose phosphate pathway (PPP) (**C**), and the tricarboxylic acid (TCA) cycle (**D**). Red coloring indicates metabolites or enzymes upregulated in PLXC1-/- MΦs, green indicates upregulation in PLXC1^+/+^ MΦs, and blue indicates metabolites or enzymes that were not measured in this study. Samples were pooled from 3 to 4 mice per group. Significance was determined by the unpaired two-tailed Student’s t-test, *P < 0.05; **P < 0.01; ***P < 0.001

To determine more precisely whether this alteration in MΦs is associated with modifications in cellular metabolism, we examined the extracellular acidification rates (ECARs) – which are a measure of lactic acid production – and oxygen consumption rates (OCRs), in peritoneal MΦs^PLXC1+/+^ and MΦs^PLXC1−/−^ following pretreatment with zymosan-A (ZyA) for 12 h. As indicated in Fig. 2A, increased basal ECARs and reduced mitochondrial OCRs (Fig. 2B) were observed in peritoneal MΦs^PLXC1−/−^, which correlated with decreases in maximal respiration and mitochondrial ATP production compared to those in MΦs^PLXC1+/+^ controls. These results suggest that PLXC1 expression deficiency shifts MΦ metabolism toward aerobic glycolysis and reduces oxidative phosphorylation (OXPHOS) (Fig. 2B). For deeper insight and understanding into the metabolic programs affected by PLXC1, NMR-based quantitative metabolomic analysis and PCR analysis of glucose metabolism were performed. In line with the Seahorse data, we observed increased glycolysis in MΦs^PLXC1−/−^, as evidenced by increased expression of key glycolysis-related genes and which was ultimately reflected in a pronounced increase in lactate concentration (Fig. 3A and B and Suppl. Table 1). Increased glycolysis was correlated with the pentose phosphate pathway (PPP) induction - which is known to promote proinflammatory responses in MΦs [14] as evidenced by the expression of glucose-6 phosphate dehydrogenase (G6PD), ribulose-3 phosphate epimerase (RPE), transaldolase (TALDO), and transketolase (TKT) (Fig. 3C). When we then focused on the TCA cycle, which has been proven to be a crucial contributor to the balance of cellular metabolism, we detected changes in key metabolites in MΦs^PLXC1−/−^ (Fig. 3D). A significant intracellular increase of itaconate was identified, which in turn resulted in enhanced inhibition and consequently, the accumulation of SDHb, a well-known inflammatory mediator in MΦs. Indeed, we found increased consumption of intracellular succinate in MΦs^PLXC1−/−^, indicating two aspects: (I).: MΦs^PLXC1−/−^ abandon the TCA cycle as a primary energy source and resort to catabolic pathways such as aerobic glycolysis, as described above. (II): The strong increase in extracellular succinate content in the cell supernatant reflected a possible additional systemic immunoregulatory role (outside the cell). Similarly, the decreased citrate levels in MΦs^PLXC1−/−^ corresponded with a lipid mediator profile where PGD_2_ and PGE_2_ were greatly reduced in PLXC1^−/−^ peritoneal exudates, indicating a lessened effect on lipid mediator class switching and ultimately the formation of SPM such as maresin-1 (Mar1) and protectin DX (PDX) (Fig. 5C and E and Suppl. Tables 1 and 2).

Taken together, these data indicate that activated MΦs^PLXC1−/−^ exhibit a bioenergetic/metabolic shift toward a more energetic and catabolic phenotype with a propensity for aerobic glycolysis promoted by impaired TCA metabolism. These results are in line with the data presented in Fig. 1.

### mTOR and AKT2 phosphorylation-signaling pathways are crucial determinants of activated MΦs^PLXC1−/^

**Fig. 4:**
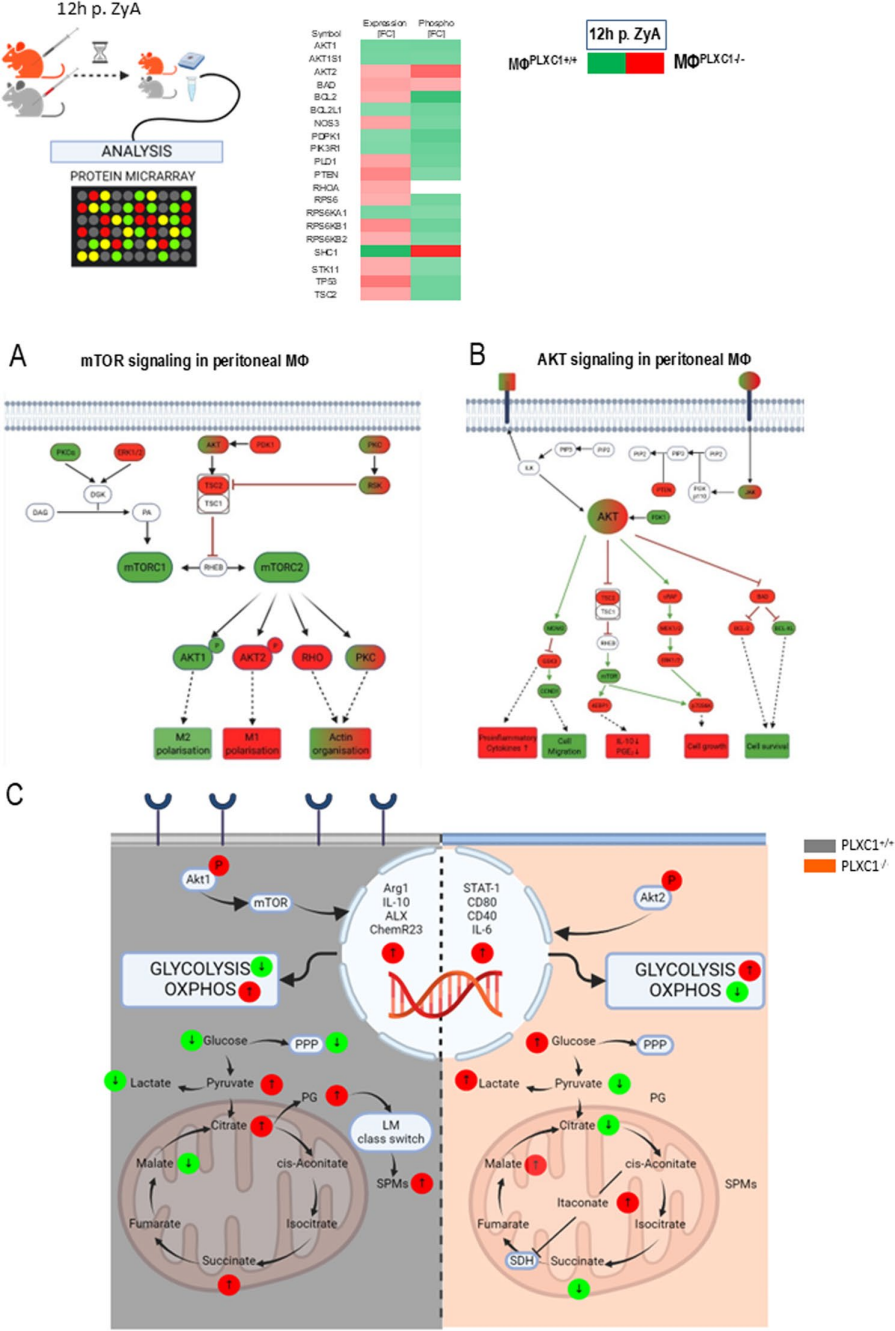
PLXC1 Deficiency Modulates Downstream Signalling Pathways in MΦ. **A** Peritoneal MΦs from PLXC1^+/+^ and PLXC1^-/-^ mice were stimulated with ZyA for 12 h. Protein microarray analysis of the mTOR (**A**) and AKT (**B**) signalling in PLXC1^+/+^ and PLXC1^-/-^ MΦs. **C** A schematic illustration of PLXC1 function during ZyA peritonitis (left: PLXC1^+/+^, right: PLXC1^-/-^). Samples were pooled from 4 mice per group. Red colouring indicates upregulation in PLXC1^-/-^, green indicates upregulation in PLXC1^+/+^

Having demonstrated that activated MΦs^PLXC1−/−^ affect metabolism via catabolic pathways, we next aimed to investigate the downstream signaling. For this purpose, peritoneal MΦs^PLXC1−/−^ were collected and stimulated with ZyA for 12 h, after which a protein microarray was performed. The collected data showed that mTOR (primarily mTORC2) activation and AKT1 phosphorylation signaling pathways were induced in MΦs^PLXC1+/+^, which consequently resulted in a proresolving M2 phenotype (Fig. 4AC). In MΦs^PLXC1−/−^, particularly increased phosphorylation of the AKT2 signaling pathway induced an M1 MΦ-phenotype, leading to an increase in proinflammatory cytokines and a decrease in anti-inflammatory/proresolving IL-10 (Fig. 4A-C and Suppl. Figure 1). Moreover, we could show particular significance in MΦs^PLXC1+/+^ is the activation of Axl, Mer/Sky, and the phosphorylation of Rac1—checkpoints that are essential for the recognition and uptake of apoptotic cells. In addition, RhoA is activated in MΦs^PLXC1−/−^, whereas it is repressed in MΦs^PLXC1+/+^, indicating that Plexin C1-mediated suppression of RhoA facilitates efferocytosis and phagocytosis (Suppl. Figure 1B, Suppl. Dataset 1). These findings show that PLXC1 acts synergistically on these factors, establishing a pro-efferocytic/phagocytic signaling profile. Thus, PLXC1 orchestrates the effective removal of apoptotic cells and ensures the proper execution of an efficient inflammatory resolution program (Suppl. Figure 6). Our protein array data provide complementary mechanistic support. Specifically, we observed a reduced expression of integrin β1 in PLXC1-deficient macrophages (MΦ^PLXC1−/−^). β1-Integrin signaling is known to regulate MΦ adhesion, migration, and polarization, and its modulation by semaphorin-receptor interactions has recently been described in the context of SEMA7A/ITGB1-dependent MΦ reprogramming [15]. Reduced β1-integrin expression in our data therefore supports a mechanism in which PLXC1 cooperates with β1-integrin to maintain balanced MΦ activation and resolution.

Together with the transcriptional alterations of “find-me” and “don’t-eat-me” receptors and the decreased phagocytic and efferocytic capacity (Fig. 1E, Supplementary Fig. 7C), these findings indicate that PLXC1 modulates macrophage phenotype and pro-resolving functionality through β1-integrin-associated signaling pathways.

### Deficiency of PLXC1 expression impaired the resolution processes in murine peritonitis

**Fig. 5:**
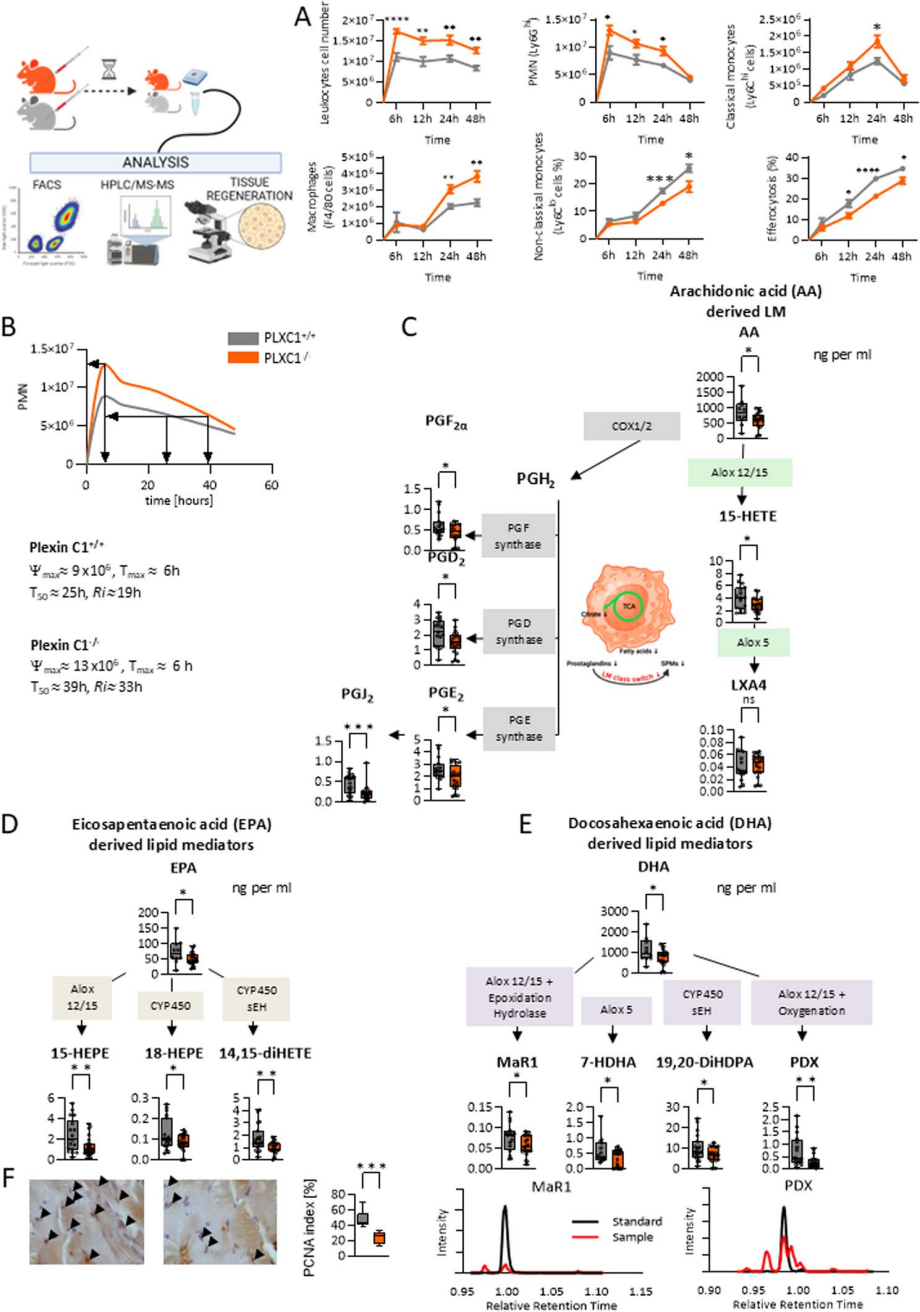
PLXC1 Deficiency Impairs Inflammation Resolution Programs. PLXC1^-/-^ mice and their littermates were injected with ZyA, and peritoneal lavages were collected at 4, 12, 24, and 48 h to assess cell dynamics during acute inflammation. **A** Total leukocytes were counted by light microscopy, and PMNs, classical Ly6Chi monocytes, F4/80^+^MΦs, non-classical monocytes, and phagocytosis of apoptotic cells were assessed by flow cytometry. **B** Resolution indices were determined. Lipid mediator profiling via LC-MS/MS analysis of peritoneal lavages from PLXC1^-/-^ mice after treatment with zymosan A for 4 h derived from Arachidonic Acid (AA) (**C**), Eicosapentaenoic Acid (EPA) (**D**), and Docosahexaenoic Acid (DHA) (**E**) was conducted (all in *ng/mL*) (*n* = 8–16). **F** Proliferating cell nuclear antigen (PCNA) expression in peritoneal slices (24 h after ZyA injection) was detected by immunohistochemistry (40x magnification, scale bar = 50 μm, *n* = 4), and the calculated indices were compared (left: PLXC1^+/+^, right: PLXC1^-/-^). The results represent at least three independent experiments and are shown as mean ± SEM (A) or as box-and-whisker plots (C–F). Panels C–E display all individual data points, with whiskers indicating the minimum and maximum values, whereas panel F shows whiskers representing the 5th–95th percentiles. Significance was determined by the unpaired two-tailed Student’s t-test, *P < 0.05; **P < 0.01; ***P < 0.001

Next, we investigated whether the proresolving aspects of PLXC1 observed in vitro could also be detected in murine ZyA-induced peritonitis. PLXC1^−/−^ mice and their littermates were injected with ZyA and peritoneal lavages were collected after 4, 12, 24 and 48 h to determine the influence of PLXC1 on cell dynamics in the different phases of acute inflammation. Compared to control mice, the PLXC1^−/−^ mice showed an increase in leukocytes and further differentiation of these cells displayed a significant increase in PMNs, classical Ly6C^hi^ monocytes and F4/80^+^ MΦs. In addition, in PLXC1^−/−^ mice the key feature of inflammation resolution was a significant reduction in non-classical monocytes which was associated with reduced efferocytosis of apoptotic cells (Fig. 5A and Suppl. Figure 2). Analysis of the resolution index indicated that the resolution interval was prolonged from 19 h in PLXC1^+/+^ mice to 33 h in PLXC1^−/−^ mice, indicating a delay in the resolution of inflammation (Fig. 5B). In resolution processes, endogenous SPMs derived from polyunsaturated fatty acids, e.g., lipoxins, resolvins, protectins and maresins, play a crucial role. Prostaglandins (PGs) orchestrate multiple roles, acting as key inflammation inducers, pro-resolvers by stimulating SPM biosynthetic enzymes, and mediators of anti-inflammatory and immunosuppressive effects. Reduced citrate in PLXC1^−/−^ peritoneal exudates correlated with decreased PGD2, PGE2, and LTB4, leading to impaired class switching and diminished SPM formation (Mar1, PDX). We generated a lipid mediator profile by investigating the expression of lipid mediators in the peritoneal lavages using a liquid chromatography-tandem mass spectrometry (LC-MS/MS)-based analysis. The data obtained from the lavages of PLXC1^−/−^ mice revealed significantly decreased levels of lipoxygenase and cytochrome-derived metabolites, 15-HETE, 15-HEPE, 18-HEPE, 14,15-diHETE, 7-HDHA, and 19,20d-DiHDPA and their metabolites Mar1 and PDX – which are associated with alterations in the related signaling pathways (Fig. 5C and E and Suppl. Tables 3 and 4). These results corroborated the MΦ metabolomics data (Fig. 3D) in which citrate was found to be less produced in MΦs^PLXC1−/−^, indicating reduced production of lipid mediators and in particular SPMs. The administration of anti-PLXC1 antibody (Suppl. Figure 4) revealed a similar inflammatory pattern, both in cell dynamics (Suppl. Figure 3A) and in the composition of lipid mediators (Suppl. Figure 3B). Finally, PLXC1 was also shown to affect the late resolution/regeneration phase by reducing the proliferating nuclear antigen (PCNA) response in PLXC1^−/−^ peritoneal tissues, which may indicate impaired tissue regeneration (Fig. 5F).

### PLXC1 is clinically correlated with the outcomes of critically ill pediatric patients with abdominal compartment syndrome

**Fig. 6:**
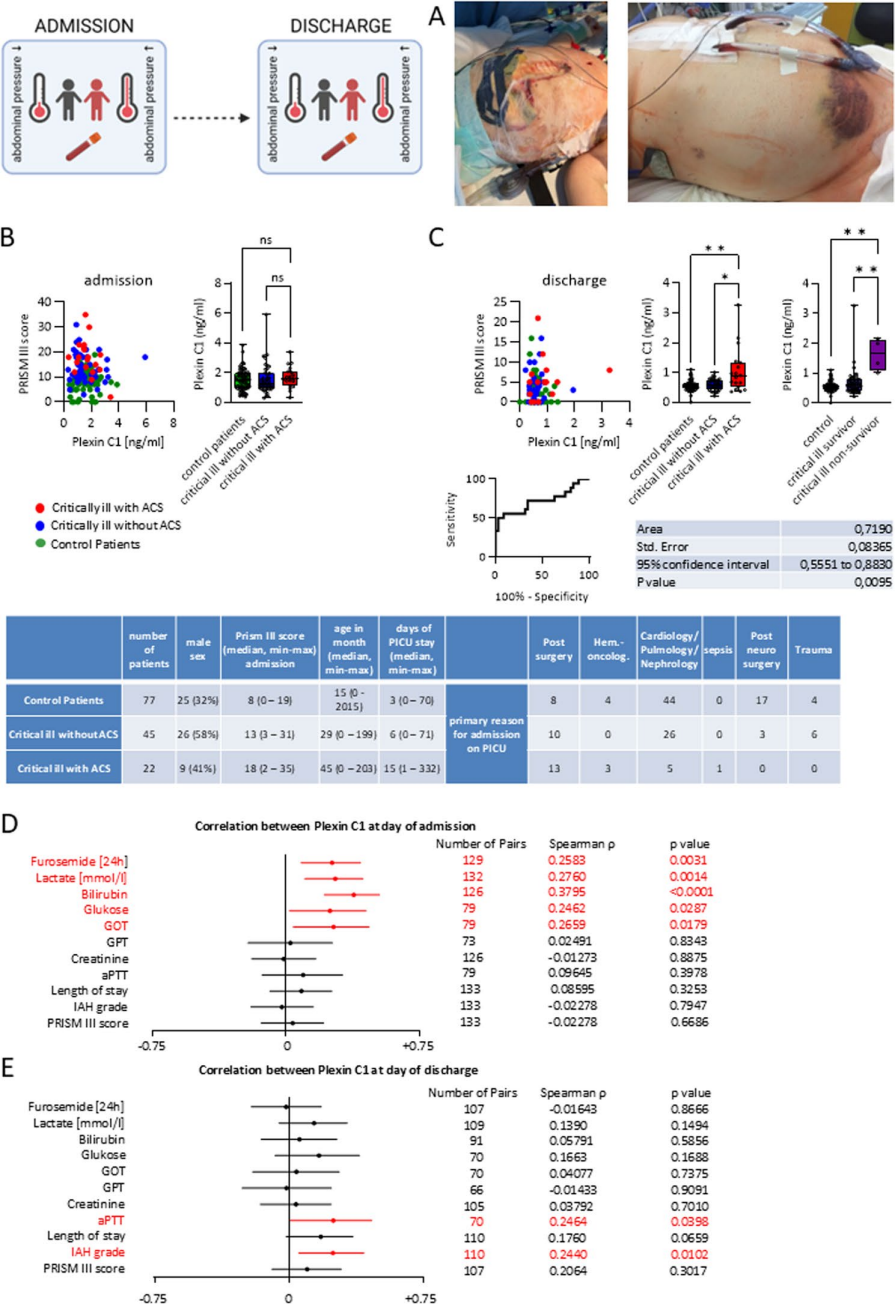
Association of Plasma PLXC1 Expression with Disease Progression in Critically Ill Children with Abdominal Hypertension. Plasma samples from 143 children in the ICU with and without abdominal compartment syndrome (ACS) were collected within 24 h after admission and on the day of discharge from the Pediatric Intensive Care Unit (PICU) of Hannover Medical School (MHH, Germany). **A** Representative images display a patient with ACS (after decompressive laparotomy with the establishment of an open abdomen/laparostoma to reduce intra-abdominal pressure (IAP)) (left) and a patient with a protuberant abdomen in intra-abdominal hypertension (right). (B-C) PLXC1 levels in control patients, critically ill patients without abdominal compartment syndrome, and critically ill patients with abdominal compartment syndrome were measured by ELISA. **B** On the day of ICU admission: an overview of the distribution of PLXC1 levels and corresponding PRISM III scores (left) and corresponding bar graphs (right). **C** On the day of ICU discharge: an overview of the distribution of PLXC1 levels and corresponding PRISM III scores, bar graphs, classification based on survival, and the corresponding area under the ROC curve (from left to right). Correlation between PLXC1 and the clinical parameters of patients in the PICU on the day of admission (**D**) and discharge (**E**) was assessed. Spearman’s rank correlation coefficient Rho and the corresponding 95% CI interval are shown (**D**, **E**). The results are expressed as box-and-whisker plots showing all individual data points (**B**, **C**); non-parametric Mann-Whitney test; correlation was tested using the Spearman’s rank correlation test; *P < 0.05, **P < 0.01, ***P < 0.001

In a population of 143 critically ill children aged < 18 and younger affected by abdominal hypertension and, depending on disease severity, abdominal compartment syndrome (ACS), we investigated a possible association between plasma PLXC1 expression in these patients and disease progression upon admission and discharge from the pediatric intensive care unit (PICU) (Fig. 6A and B). Vital signs, cardiopulmonary parameters, medication administration, fluid balance and intra-abdominal pressures were constantly monitored.

We divided the patients into critically ill patients with and without ACS. The group of critically ill children with high intra-abdominal pressure tended to have higher PLXC1 plasma levels upon admission to the PICU than did the control group. Interestingly, there was a significant correlation between PLXC1 expression and lactate levels, even in the earlier phase of inflammation. This indicated that there was likely a translocation of PLXC1 to the extracellular space, where it was strongly secreted for the activation of inflammation resolution processes. Intracellularly, immunometabolic processes such as aerobic glycolysis were consequently activated as compensation (Fig. 6D and E). These data are consistent with the immunometabolism data (Fig. 3A-B). However, on the day of discharge from the PICU, the group of critically ill ACS patients displayed significantly greater plasma levels of PLXC1 than did the control group and the group of patients without ACS; the increased plasma PLXC1 levels were also associated with significantly greater mortality. This is an indication that the inflammatory resolution processes failed, leading to the accumulation of PLXC1 expression. As a result, PLXC1 could be used as a potential biomarker or predictor of morbidity and mortality in critically ill pediatric patients with severe inflammation (Fig. 6C).

## Discussion

The exacerbation of excessive inflammatory signals and the orchestration of a wide variety of inflammatory cells may lead to a cycle of dysfunction and subsequent dysregulation. These so-called nonresolving inflammatory processes are the cause of severe diseases such as sepsis or ARDS and remain a major therapeutic challenge, rendering the investigation of ways to improve inflammatory resolution an unmet clinical need [16]. Although it was previously considered a passive process, the resolution of acute inflammation is in fact an active and highly coordinated program driven and controlled mainly by endogenous immunoresolvents such as specialized proresolving lipid mediators (SPMs) [10]. At the cellular level, cells of monocyte/macrophage (MΦ) lineage in particular guide this process, by monitoring tissue integrity and thus playing a major role in tissue homeostasis and repair [11, 10, 17]. Specifically, the heterogeneity and plasticity of these cells enables them to perceive the microenvironment and adapt their attributes accordingly [18]. New findings have shown that the homeostatic pathways of immune cells are altered during the inflammatory process, leading to a complex interplay between metabolic reprogramming and immunity and ultimately revealing therapeutic potential [19]. 

Over the past years, NGPs have garnered significant interest for their capacity to modulate inflammatory responses. Their effects in the initial phase of inflammation show exciting insights, in contrast, we are in the pioneer phase regarding their influence on inflammation resolution and tissue regeneration [3, 4]. Since inflammation resolution is characterized by dynamic changes in cell migration and cell compositions, the effects of NGPs are highly heterogeneous. The dependent variables are the phases of the inflammatory process, the involvement of specific immune cell types and the affected systems or organs. Thus, for instance, PLXC1 has a proinflammatory effect in the initial phase of LPS-induced lung inflammation [8] or liver ischemia-reperfusion injury [20], while it provides an anti-inflammatory effect on osteoarthritis [21]. In this study, we showed that PLXC1 has proresolving impact in the context of murine peritonitis.

It is clear that ligands play an important role in this respect. Other reasons for the contrasting effects of PLXC1 may include the strength of its affinity to its ligand, or the extent of receptor expression in tissues or immune cells and competition with coreceptors. For example, Sema7A is a ligand of PLXC1 and Sema7A also interacts with a group of integrins [22]. More recently, Bernard et al. demonstrated PLXC1 to suppress excessive noncanonical inflammasome activity and thus improving survival during CLP- induced sepsis.

Proresolving control mechanisms are activated by the onset of acute inflammation as early as the transition phase, and the characteristics of whether proinflammatory processes lead to resolution and tissue repair or persist have become increasingly clear [23]. PMN apoptosis, in particular, plays an effective role in this process, as apoptotic PMNs send soluble signals to attract MΦs and thus activate efferocytosis/phagocytosis [24]. Although a remarkable number of molecules and mechanisms have been described in the efferocytosis/phagocytosis of apoptotic immune cells, in this work we showed that stimulation of human MΦs with an anti-PLXC1 ab reduced the expression of three crucial receptors - CX3CR1, TIM4 and P2Y2 – all of which are related to “find me” signaling. Subsequently the expression of “don’t eat me” receptor SIRPα was significantly increased. There is no doubt that MΦs are key players in processes of inflammation and inflammation resolution owing to their multifunctional capabilities and heterogeneity [18]. Due to their plasticity, flexibility and activation states, MΦ are most likely to be the most adaptable cells among cells of hematopoietic origin. The classification into two main functional phenotypes, the proinflammatory M1 and the anti-inflammatory/proresolving M2 type reflects the extremes of a broad spectrum of differentiation states [25, 26]. Thus, our data show that stimulation with anti-PLXC1 ab alters MΦ polarization to a proinflammatory phenotype, characterized by an increase in the M1-related markers CD40, CD80 and STAT-1 and a reduction in the M2-related marker arginase. These results were corroborated by the increase in proinflammatory IL-6 and decreases in IL-10 and the specific SPM receptors ALX and Chem23, indicating the proresolving impact of PLXC1. In recent years, the metabolic reprogramming has gained special significance in research regarding tumors and especially inflammation. Proinflammatory immune cells acquire their primary energy source following activation via catabolic metabolic pathways such as aerobic glycolysis, and not through the TCA cycle. The metabolites derived from the TCA cycle and glycolysis not only undergo the metabolic transformations in immune cells via bioenergetics and biosynthesis, but also change their characteristics through certain effector mechanisms [19, 27]. In line with this research, we detected an increase in the extracellular acidification rate (ECAR) and a decrease in the mitochondrial oxygen consumption rate (OCR), maximal respiration and mitochondrial ATP production in peritoneal MΦs^PLXC1−/−^, following pretreatment with ZyA, thus reflecting activation of the M1 phenotype. The in-depth analyses of MΦs^PLXC1−/−^ metabolic programs revealed increased expression of key glycolysis-related genes and finally a significant increase in lactate production compared to those in control MΦs^PLXC1+/+^. This phenomenon correlated with induction of the pentose phosphate pathway (PPP), which is known to drive proinflammatory responses in MΦs [14]. Furthermore, we detected changes in mitochondrially generated tricarboxylic acid (TCA) cycle metabolites in MΦs^PLXC1−/−^. A significant intracellular increase in the intracellular concentration of itaconate was observed, resulting in the enhanced inhibition and subsequent accumulation of SDHb, a widely recognized MΦ-related inflammatory mediator. In fact, we observed an increased demand for intracellular succinate in MΦs^PLXC1−/−^, demonstrating two different perspectives: (I): MΦs^PLXC1−/−^ abandon the TCA cycle as a primary energy source and resort to catabolic pathways such as aerobic glycolysis. (II): The strong elevations of extracellular succinate in the cell supernatant suggest a possible further systemic immunoregulatory role. The intracellular and extracellular effects of succinate appear to be divergent [19]. Interestingly, Littlewood-Evans et al. described an extracellularly released SUCNR1/succinate interaction in leukocyte activation, inducing further IL-1β production [28]. Keiran et al. showed that activation of SUCNR1 promotes an anti-inflammatory phenotype in MΦs. In addition, in our study decreased citrate levels were detected in MΦs^PLXC1−/−^, which correlated with a lipid mediator profile in which PGD2 and PGE2 were greatly reduced in the peritoneal exudates of PLXC1^−/−^ mice. This suggests a lower effect on lipid mediator class switching and ultimately the formation of SPMs such as Mar1 and PDX. At the signaling level, we showed that in activated MΦs^PLXC1−/−^ the switch to catabolic metabolism and thus to the M1 phenotype is induced by the activation of mTOR and, in particular, by increased phosphorylation of the AKT2 signaling pathway.

In addition to the important role that immune cells such as monocytes and MΦs play in the inflammation resolution process, where they serve as sensors and/or as effectors, mediators – the so-called immunoresolvents - play an elementary role as control elements [1, 16, 29]. There is broad consensus that the endogenous classes of SPMs serve as key modulators of immune function. Both SPMs (e.g. lipoxins, resolvins, protectins and maresins) and their metabolites are locally generated during an inflammatory process and stimulate important cellular and molecular steps of resolution to return the tissue to a homeostatic state [10]. In this work we demonstrated that in a murine ZyA-induced peritonitis model, PLXC1^−/−^ mice exhibited an increase in leukocytes, which in addition to further differentiation, displayed a significant increase in PMNs, classical Ly6C^hi^ monocytes and F4/80^+^ MΦs in a time-dependent manner and a significant reduction in non-classical monocytes associated with reduced efferocytosis of apoptotic cells. In addition, we observed decreased levels of Mar1 and PDX and their pathway markers − 15-HETE, 15-HEPE, 18-HEPE, 14,15-diHETE, 7-HDHA, and 19,20d-DiHDPA, supporting our hypothesis that PLXC1 could be considered an immunoresolvent. Moreover, further classes of mediators have been identified as immunoresolvents [29]. NGPs, which may possess autonomous proresolving properties play an interesting role. They may activate the formation of SPMs and synergically exhibit a stronger effect on inflammation resolution processes [30–32]. Nevertheless, how these mediators interact with each other and what impact they have on therapy as immunoresolvents in severe diseases such as sepsis or ARDS in humans have not yet been sufficiently described. However, it is quite evident that in light of accumulating evidences, there is a paradigm shift in our thinking from “fighting inflammation” to “promoting inflammation resolution”. Further research will be needed in the upcoming years.

Beyond the mRNA analyses and protein pathway profiling performed, additional validation of AKT2 signaling at the protein or functional level may provide further mechanistic insight and strengthen the overall interpretation of the data.

In conclusion, these findings reveal a novel role for PLXC1 in regulating the crosstalk between immune metabolism and inflammation resolution programs in severe inflammation and may pave the way for new therapeutic approaches.

## Materials and methods

Additional material and methods are provided in the Supporting Information (Supplementary Material 1 (Supporting Information) ).

### Animals

This project was approved by the Institutional Review Board and Regierungspräsidium Tübingen. C57BL/6J mice were purchased from Charles River Laboratories (Sulzfeld, Germany) and used for exogenous application of the anti- PLXC1 antibody treatment.

Plexin C1 Knockout *(PLXC1*^*−/−*^*)* mice on a *C57BL/6J* background and littermate control mice (PLXC1 ^+/+^) were bred and genotyped as previously described [8].

### Zymosan A peritonitis

Animal protocols were conducted in compliance with the regulations of the Regierungspräsidium Tübingen and the local ethics committee. Mice received an intraperitoneal (i.p.) injection of 1 ml of ZyA (1 mg/ml; Sigma-Aldrich). In a subgroup, mice additionally received either a vehicle (Mouse IgG2b) or 1 µg of the anti-PLXC1 antibody (MAB3887, Monoclonal Mouse IgG2B Clone # 544232, R&D Systems, Inc., Minneapolis, USA) in a total volume of 150 µl intravenously via tail vein injection. Peritoneal fluids and tissues were collected at 4, 12, 24, and 48 h and prepared as previously described.

### Data analysis

Comparative data analysis was conducted using either one-way ANOVA or unpaired two-tailed Student’s t-test, depending on the specific experimental design, as detailed in the figure legends. A p-value of less than 0.05 was considered to indicate statistical significance. The exact number of biological replicates (independent experiments or animals) is specified in each figure legend. Data are presented as box-and-whisker plots displaying all individual data points, with whiskers indicating minimum and maximum values, unless otherwise stated. All statistical analyses were performed using GraphPad Prism (version 10; GraphPad Software, San Diego, CA, USA).

### Human leukocyte isolation and macrophage differentiation and polarization

Human peripheral blood monocytes (PBMCs) were isolated from healthy volunteers or from leukapheresis collars provided by the Blood Bank of Eberhard Karls University of Tübingen. For differentiation experiments, cells were cultured in RPMI 1640 medium supplemented with 10% fetal calf serum (FCS), 1% penicillin/streptomycin, and either 10 ng/ml human recombinant GM-CSF (Macs Milteny, Bergisch Gladbach, Germany), or 20 ng/ml M-CSF (Macs Milteny, Bergisch Gladbach, Germany) at 37 °C for 7 days. For polarization, M1 macrophages (MΦ) (cultured with GM-CSF for 7 days) were stimulated with 10 ng/ml IL-1β (R&D Systems, Inc., Minneapolis, USA) ± 1 µg of anti-PLXC1 ab for 24 h, followed by analysis.

### RNA extraction, cDNA synthesis, and quantitative PCR analysis

Total RNA was extracted from cultured cells using TRIzol reagent (Invitrogen, USA) according to the manufacturer’s instructions. The RNA concentration and purity were assessed spectrophotometrically. cDNA was synthesized from 500 ng of total RNA using the iScript™ cDNA Synthesis Kit (Bio-Rad, USA). Quantitative real-time PCR was performed using gene-specific primers and SYBR Green chemistry on a CFX96™ Real-Time PCR Detection System (Bio-Rad, USA). mRNA expression levels were normalized to 18 S using the ΔΔCq method. All reactions were run in duplicate, and no-template controls were included to check for contamination.

### Analysis of macrophage phenotype and receptor expression

Transcriptional analysis of human Plexin C1 mRNA expression was conducted using the sense primer 5’- GAT ACG TGA CGG CTT TGC TG −3’ and antisense primer 5’- AGT GAA GAT GTG GGT GAA GCC − 3’. Human 18S expression was evaluated with the sense primer 5’-GTA ACC CGT TGA ACC CCA TT-3’ and antisense primer 5’- CCA TCC AAT CGG TAG TAG CG-3’. The following primers were used to determine MΦ phenotype: CD80: 5’- AGC CTC ACC TCT CCT GGT TG-3’, 5’- TGG GGC AAA GCA GTA GGT CA − 3’; STAT-1: 5’- ATC AGG CTC AGT CGG GGA ATA-3’, 5’- TGG TCT CGT GTT CTC TGT TCT − 3’; CD40 5’- ACT GAA ACG GAA TGC CTT CCT-3’, 5’- CCT CAC TCG TAC AGT GCC A − 3’; Arg1: 5’- TGG ACA GAC TAG GAA TTG GCA − 3’, 5’- CCA GTC CGT CAA CAT CAA AAC T − 3’; IL-6: 5’- GAC AGC CAC TCA CCT CTT CA − 3’, 5’- CAC CAG GCA AGT CTC CTC AT − 3’; IL-10: 5’- AAT CGA TGA CAG CGC CGT AG − 3’, 5’- GGT TGC CAA GCC TTG TCT GA − 3’; P2Y_2_: 5’- CCG CTT CAA CGA GGA CTT CAA − 3’, 5’- GCG GGC GTA GTA ATA GAC CA − 3’; CX_3_CR_1_: 5’- AAT GCC TGG CTG TCC TGT GT − 3’, 5’- GCC TGC TCC TTT GTG ATT CAG − 3’; TIM4: 5’- ACA GGA CAG ATG GAT GGA ATA CCC − 3’ and SIRPα: 5’- ATA CGC AGA CCT GAA TGT GCC CAA − 3’, 5’ – TGG CCA CTC CAT GTA GGA CAA GAA – 3’. Transcriptional analysis of G protein-coupled receptors (GPCRs), such as ALX/FPR2 and ChemR23, which mediate pro-resolving actions, was performed using the following primers: ALX/FPR2: 5’- TGT TCT GCG GAT CCT CCC ATT- 3’, 5’- CTC CCA TGG CCA TGG AGA CA- 3’. ChemR23: 5’- AGG GAC TGA TTG GCT GAG GA − 3’, 5’- ATC CTC CAT TCT CAT TCA CCG T − 3’.

### Human macrophage phagocytosis/efferocytosis

GM-CSF-differentiated MΦ (0.1 × 10^6 cells/well) were incubated with anti-PLXC1 ab or IgG control as indicated. Fluorescent ZyA particles (Molecular Probes, Z-2841) were added at a 1:10 ratio (macrophages: ZyA) and incubated at 37 °C for 90–120 min to allow phagocytosis. After incubation, cells were washed thoroughly with PBS to remove unbound particles. In parallel, Zymosan-only control wells (identical particle concentration and handling, but without cells) were measured to account for background fluorescence. For complementary assays with apoptotic cells, Green Cell Tracker Dye (Cat. No. C7025; Invitrogen)-labelled apoptotic PMNs were co-incubated with macrophages at a 1:3 (MΦ:PMN) ratio for 90 min at 37 °C, followed by imaging and quantification of internalized cells. Fluorescence was measured using a fluorescent plate reader (Tecan, Männedorf, Switzerland), and imaging of phagocytotic activity was performed using Confocal microscope (Leica, Stellaris 8, Germany).

### Differential leukocyte counts, flow cytometry analysis, and ELISA

To assess cellular composition, exudate cells were isolated from a murine peritonitis model. Initially, cells were blocked with mouse anti-CD16/CD32 antibodies for 10 min at room temperature. Following this, cells were stained with anti-mouse APC-Ly6G, e450-F4/80, and FITC-Ly6C antibodies (all from Biolegend) for 30 min at 4 °C. For the in vivo analysis of MΦ phagocytosis of apoptotic PMNs, cells underwent permeabilization and subsequent staining with PerCP-Cy5.5-conjugated anti-Ly6G (Biolegend) for 30 min at 4 °C. Flow cytometry analysis was performed on a BD FACSCanto II. Additionally, cytokine levels in murine peritoneal exudates were quantified using standard ELISA kits (R&D Systems).

### Pediatric ICU patient samples with and without abdominal compartment syndrome (ACS)

Plasma samples were obtained from 143 pediatric patients in the Pediatric Intensive Care Unit (PICU) at Hannover Medical School (MHH, Germany). Collections occurred within 24 h of admission and again on the day of discharge. The 2013 WSACS definitions [33] for intra-abdominal pressure (IAP) and abdominal compartment syndrome (ACS) (www.wsacs.org) were applied to diagnose ACS. The severity of illness in these patients was assessed using the PRISM III [34] scoring system. Continuous recording of vital signs, cardiorespiratory parameters, drug administration, intra-abdominal pressures (measured using the gastric Spiegelberg^®^ monitoring system [35]), and fluid balances was maintained through the digital patient data management system (mlife, mediside). PLXC1 levels were measured using an ELISA kit (CUSABIO, # CSB-EL018225HU), following the manufacturer’s protocol.

### LC-MS/MS

Peritoneal lavage samples were supplemented with 4 µL of an internal standard solution (comprising PGE4-d4, LTB4-d4, 15-HETE-d8, and DHA-d6 at a concentration of 50 ng/ml in methanol). The samples were then transferred to 12-ml glass vials, to which 1.75 ml of methanol was added. Following centrifugation at 4,000 rpm for 5 min at 6 °C, the supernatant was transferred to new 12-ml glass vials. The remaining pellet was re-extracted with 500 µL of methanol and centrifuged again under the same conditions. The organic extracts were combined, and methanol was partially evaporated under a gentle stream of nitrogen at 40 °C for 30 min. The resulting methanolic extract (approximately 1.5 mL) was then diluted with 8 ml of water, and 20 µL of 6 M HCl was added. The prepared samples underwent solid phase extraction (SPE) using SepPak C18 200 mg cartridges (Waters, MA, USA). The SPE cartridges were preconditioned with 2 mL of methanol followed by 2 mL of water. The samples were loaded, washed with 3 mL of water, followed by 3 mL of n-hexane, and then eluted with 3 mL of methylformate. The eluate was dried under a gentle stream of nitrogen, reconstituted in 200 µL of 40% methanol, and injected for analysis.

For the LC-MS/MS analysis, a QTrap 6500 mass spectrometer operating in negative ESI mode (Sciex, Nieuwerkerk aan den IJssel, The Netherlands) was used, coupled to an LC system featuring two LC-30AD pumps, a SIL-30AC autosampler, and a CTO-20AC column oven (Shimadzu, ‘s-Hertogenbosch, The Netherlands). The chromatographic separation was performed on a 1.7 µm Kinetex C18 50 × 2.1 mm column with a C8 precolumn (Phenomenex, Utrecht, The Netherlands), maintained at 50°C. A binary gradient composed of water (solvent A) and methanol (solvent B) with 0.01% acetic acid was employed as follows: starting at 30% B for 1 minute, ramping to 45% B at 1.1 minutes, 53.5% B at 2 minutes, 55.5% B at 4 minutes, 90% B at 7 minutes, 100% B at 7.1 minutes, and maintained for 1.9 minutes. The injection volume was 40 µL, with a flow rate of 400 µL/min. Analyte identification was achieved by combining specific mass transitions of each analyte with its relative retention time (RRT). Calibration lines constructed from standard materials for each analyte were utilized for quantification, and only peaks with a signal-to-noise (S/N) ratio greater than 10 were quantified.

### Metabolic extracellular flux assay

Peritoneal MΦs were isolated and seeded into XF-96 cell culture plates (1 × 10^6 cells per well) (Seahorse Bioscience) according to the experimental requirements. Non-adherent cells were removed by washing once with PBS. To evaluate metabolic programs, the Seahorse XF Glycolysis and Mito Stress Tests were performed as per the manufacturer’s instructions. Extracellular acidification rates (ECARs) and oxygen consumption rates (OCRs) were measured using an XF-96 Flux Analyzer (Seahorse Bioscience). Glycolysis was quantified by observing ECAR changes in response to the injection of glucose (final well concentration (fwc): 10 mM), oligomycin (OM; fwc: 1.0 µM), and 2-deoxy-D-glucose (2-DG; fwc: 50 mM). Oxidative phosphorylation (OXPHOS) characteristics were determined from OCR changes in response to OM (fwc: 1.0 µM), carbonyl cyanide-p-trifluoromethoxyphenylhydrazone (FCCP; fwc: 2.0 µM), and rotenone plus antimycin A (ROT + AA; fwc: 0.5 µM). For normalization of results, cells were washed once with PBS, fixed with 4% paraformaldehyde (PFA) containing Hoechst 33,342 for 5 to 10 min, and then analyzed for fluorescence using a fluorescence plate reader to quantify nuclear staining as a direct measure of cell number.

### NMR spectroscopy and quantification of metabolites

NMR analysis was done on mice samples from three independent experiments over two different labs. In lab I (University Hospital Tübingen, Germany), extracts were prepared according [36], transferred to a 1.7 mm NMR tube and measured at 298 K with a triple resonance (TXI) 1.7 mm room temperature probe at 600 MHz (Bruker Avance III HD). Recorded Carr-Purcell-Meiboom-Gill (CPMG, 4096 scans) spectra were processed and annotated with Chenomx NMR Suite 8 (Chenomx, Edmonton, Canada). In lab II (Leiden University Medical Center, Netherlands), operating a 600 MHz Bruker Avance II systesm, analysis of intracellular metabolites was conducted as previously described [37]. In brief, dried extracts were reconstituted in 250 µL of 0.15 M K2HPO4/KH2PO4 buffer (pH 7.4) in 99.9% deuterated water (D2O), containing 0.2 mM NaN3 and 0.4 mM trimethylsilylpropionic acid sodium salt (TSP-d4), and subsequently transferred to 3-mm NMR tubes. NMR data were acquired using a 14.1 T NMR spectrometer under standardized conditions for all samples. All spectra underwent phase and baseline correction and were referenced to TSP-d4. The one-dimensional (1D) spectra were imported into the Chenomx NMR Suite 8 (Chenomx, Edmonton, Canada) for quantification purposes. Metabolite identification was performed using the Bbiorefcode (Bruker Biospin) and Chenomx databases, with the structures of all annotated metabolites confirmed through two-dimensional (2D) NMR experiments on the same samples. The concentrations of quantified metabolites (in mM) were normalized to the cell count per sample. For clustering and heatmap presentation of quantitative data, the R package “factoextra” (http://www.r-project.org/, R version 3.4.4) was employed. Data from both NMR labs were pooled (Supplement Tables 1 and 2) and used for final pathway analysis.

### Antibody array for protein expression

Peritoneal monocytes/MΦs from PLXC^1−/−^ and PLXC1^+/+^ mice were collected at 12 h post-ZyA-induced peritonitis. Protein and phosphorylation profiling (mTOR Phospho Antibody Array, FullMoonBioscience, #PMT138) of these peritoneal monocytes/MΦ (pooled lavages from 4 mice per condition) was conducted following the manufacturer’s protocol. Image acquisition was performed by the manufacturer. For each antibody, the average signal intensity of 6 replicates was normalized to the median signal of all antibodies on the array. The fold change presented represents the ratio of the normalized signal in PLXC1^−/−^ mice compared to PLXC1^+/+^ mice. GAPDH and beta-actin served as housekeeping proteins. Data analysis was carried out using IPA software (Qiagen), with pathways being substantiated and updated through recent literature, as well as the KEGG (HSA 04150; HSA 04151) and Reactome (R-HSA-165159, R-HSA-198203) databases.

### Glucose metabolism RT2 profiler PCR array

Peritoneal MΦs from PLXC1^−/−^ and PLXC1^+/+^ mice were used following 0 and 12 h of ZyA treatment. RNA isolation was performed using the miRNeasy Mini Kit, and cDNA synthesis was carried out with the RT2 Easy First Strand Kit (Qiagen). Expression profiling of peritoneal MΦ (pooled lavages from 2 mice per condition) was conducted following the manufacturer’s instructions. The reported fold change reflects the ratio of normalized signals from PLXC1^−/−^ mice compared to PLXC1^+/+^ mice. GAPDH, beta-actin, beta-2 microglobulin, beta-glucuronidase, and heat shock protein 90 alpha served as housekeeping genes. Data analysis was executed using the web-based RT2 Profiler PCR Array data analysis tool and IPA software (Qiagen). Pathways were corroborated and updated using current literature.

## Supplementary Information

Supplementary Material 1; Supporting Information.

Supplementary Material 2; Supplementary Figure 1.

Supplementary Material 3; Supplementary Figure 2.

Supplementary Material 4; Supplementary Figure 3.

Supplementary Material 5; Supplementary Figure 4.

Supplementary Material 6; Supplementary Figure 5.

Supplementary Material 7; Supplementary Figure 6.

Supplementary Material 8; Supplementary Figure 7.

Supplementary Material 9; Supplementary Figure 8.

Supplementary Material 10; Supplementary Table 1.

Supplementary Material 11; Supplementary Table 2.

Supplementary Material 12; Supplementary Table 3.

Supplementary Material 13; Supplementary Table 4.

Supplementary Material 14; Supplementary Dataset 1.

## Data Availability

All data generated or analysed during this study are included in this published article and its supplementary information files.

## Abbreviations

PMN: Polymorphonuclear cell
MΦ: Macrophage
MCP-1: Monocyte chemotactic protein
ZyA: Zymosan A
AA: Arachidonic acid
SPM: Specialized proresolving lipid mediator
14, 15-EET: 14 15-epoxyeicosatrienoic acid
DHA: Docosahexaenoic acid
EPA: Eicosapentaenoic acid
HDHA: Hydroxydocosahexaenoic acid
HETE: Hydroxyeicosatetraenoic acid
19,20-DiHDPA: 19,20-dihydroxydocosapentaenoic acid
19,20 EDP: 19,20-epoxydocosapentaenoic acid
LXA_4_: Lipoxin A_4_
PDX: Protectin DX
Mar1: Maresin 1
LC‒MS/MS: Liquid chromatography tandem mass spectrometry
ECAR: Extracellular acidification rate
OCR: Oxygen consumption rate
HK2: Hexokinase 2
GAPDH: Glyceraldehyde 3-phosphate dehydrogenase
PFKL: 6-phosphofructokinase
ENO1: Enolase 1
NO: Nitric oxide
PPP: Pentose phosphate pathway
G6PD: Glucose-6-phosphate dehydrogenase
PGLS: 6-phosphogluconolactonase
RPIA: Ribulose-5-phosphate isomerase
TKT: Transketolase
TCA: Tricarboxylic acid
IDH: Isocitrate dehydrogenase
SDH: Succinate dehydrogenase
CS: Citrate synthase
