# Supplementary material for: Plexin C1 modulates metabolic programming for resolution of severe inflammation
Source: Cell Commun Signal. 2025 Nov 25;23:523. doi: 10.1186/s12964-025-02518-z (PMC12690942; doi:10.1186/s12964-025-02518-z)
Supplement: Supplementary file 1 — Supplementary Material 1; Supporting Information. [file 12964_2025_2518_MOESM1_ESM.pdf]

Supporting Information

Plexin C1 Modulates Metabolic Programming for Resolution of Severe Inflammation

Andreas Körner[1,2], Michael Koeppen[2], Jasvir Kaur[2], Julia C. Fitzgerald[3], Sarantos Kostidis[4], Torsten Kaussen[5], Christoph Trautwein[6,7], Martin Giera[4], Tamam Bakchoul[8], Alice Bernard[2], Valbona Mirakaj[1,2,9]

[1]Department of Anesthesiology and Intensive Care Medicine, Molecular Intensive Care Medicine, University Hospital Eberhard-Karls University, Tübingen, Germany
[2]Department of Anesthesiology and Intensive Care Medicine, University Hospital Eberhard-Karls University, Tübingen, Germany
[3]Hertie Institute for Clinical Brain Research, University Clinic Tübingen, Tübingen, Germany.
[4]Center for Proteomics and Metabolomics, Leiden University Medical Center (LUMC), The Netherlands
[5]Department of Pediatric Cardiology and Pediatric Intensive Care Medicine, Hannover Medical School, Hannover, Germany.
[6]Werner Siemens Imaging Center, Department of Preclinical Imaging and Radiopharmacy, Eberhard Karls University Tübingen, Germany
[7]Core Facility Metabolomics, Medical Faculty of Tübingen, University of Tübingen, Germany
[8]Transfusion Medicine, Medical Faculty of Tübingen, University Hospital of Tübingen, Germany
[9]Department of Anesthesiology, Intensive Care Medicine, and Pain, Translational Intensive Care Medicine, University Hospital Goethe University, Frankfurt, Germany

MATERIALS AND METHODS

Western Blot. Cells were lysed in RIPA buffer (Thermo Fisher Scientific) supplemented with protease and phosphatase inhibitors (Roche). Protein concentration was determined using the BCA assay (Thermo Fisher Scientific). Equal amounts of protein were separated by SDS–PAGE (10% or 12% gels) and transferred onto PVDF membranes. After blocking with 5% non-fat dry milk in TBS-T (0.1% Tween-20) for 1 hour at room temperature, membranes were incubated overnight at 4°C with the respective primary antibodies. Following washing, membranes were incubated with HRP-conjugated secondary antibodies for 1 hour at room temperature.

FIGURE LEGENDS

Suppl. Table 1: Intracellular Metabolites Evaluated by NMR Analysis. Intracellular metabolites were assessed by NMR analysis in $M\Phi^{PLXC1+/+}$ and $M\Phi^{PLXC1-/-}$ at baseline and following 12 hours of stimulation with Zymosan A (ZyA). All results are reported in micromolar (µM) and normalized to protein content. The data represent at least three independent experiments with n=6-15 mice per group and were compared using the unpaired two-tailed Student's t-test.

Suppl. Table 2: Metabolite Profiling of Supernatant Media via NMR Analysis. Metabolites in the supernatant media were evaluated by NMR analysis in $M\Phi^{PLXC1+/+}$ and $M\Phi^{PLXC1-/-}$ at baseline and following 12 hours of stimulation with Zymosan A (ZyA). All results are reported in micromolar (µM). Data are derived from a minimum of three independent experiments with n=6-15 mice per group and were analyzed using the unpaired two-tailed Student's t-test.

Suppl. Fig. 1: Differential Activation of the AKT/mTOR Pathway in $PLXC1^{-/-}$ and $PLXC1^{+/+}$ Mice. (A) Pathway analysis generated using *QIAGEN Ingenuity Pathway Analysis (IPA)* illustrating the predicted activation state of the AKT/mTOR signaling cascade based on protein array data obtained from peritoneal monocytes/macrophages of $PLXC1^{-/-}$ and $PLXC1^{+/+}$ mice 12 hours after zymosan A–induced peritonitis. The diagram highlights the expected activation and inhibition patterns of major signaling components within the pathway. **(B)** Array data showing differential phosphorylation of key efferocytic and metabolic regulators, including RhoA, Axl, Mer/Sky, AKT1 (Ser473, Thr308), and AKT2, detected using specific phospho-antibodies. Peritoneal macrophages were analyzed 12 hours post-ZyA stimulation.

Suppl. Fig. 2: FACS gating strategy for leukocyte differentiation. Leukocytes were gated based on forward scatter (FSC) and side scatter (SSC) parameters, and singlets were identified

by plotting FSC-A against FSC-H. Subsequently, leukocyte subtypes were further classified into Ly6G^{high}, Ly6C^{high}, and Ly6C^{low} populations. Macrophages (MΦ) were identified as F4/80 positive (F4/80^{+}). To define efferocytosis, the differentiation between intracellular and extracellular polymorphonuclear neutrophils (PMNs) was assessed using Ly6G-PerCP-Cy5.5 and Ly6G-APC antibodies. Phagocytized PMNs were identified as Ly6G-PerCP-Cy5.5 positive (Ly6G-PerCP-Cy5.5^{+}) and Ly6G-APC negative (Ly6G-APC^{-}).

Suppl. Table 3: Lipid Mediator Profile. Lipid mediator levels in murine peritoneal fluids following the administration of ZyA in PLXC1$^{+/+}$ and PLXC1$^{-/-}$ mice. Lavages were collected at 4 hours and 12 hours post-administration, and LC-MS/MS-based profiling was performed. The table includes levels of bioactive lipid mediators and their precursors derived from the arachidonic acid (AA), docosahexaenoic acid (DHA), and eicosapentaenoic acid (EPA) pathways. All results are reported in ng/ml. Data represent three independent experiments with n=20 mice per group and are expressed as the mean ± SEM. Statistical significance was determined using an unpaired Student's t-test.

Suppl. Table 4: Lipid Mediator Profile. Lipid mediator levels in murine peritoneal fluids following the administration of ZyA in PLXC1$^{+/+}$ and PLXC1$^{-/-}$ mice. Lavages were collected at 24 hours post-administration, and LC-MS/MS-based profiling was performed. The table includes levels of bioactive lipid mediators and their precursors derived from the arachidonic acid (AA), docosahexaenoic acid (DHA), and eicosapentaenoic acid (EPA) pathways. All results are reported in ng/ml. Data represent three independent experiments with n=20 mice per group and are expressed as the mean ± SEM. Statistical significance was determined using an unpaired Student's t-test.

Suppl Fig. 3: Anti-PLXC1 Antibody Treatment Impairs Inflammatory Resolution and Pro-Resolving Mediator Production in Zymosan-A-Induced Peritonitis. Wild-type (WT)

mice were treated with either 1 μg of an anti-PLXC1 antibody or an IgG control antibody, followed by intraperitoneal (i.p.) injection of 1 mg zymosan A (ZyA). Peritoneal lavages were collected at 4, 12, 24, and 48 hours to evaluate cell dynamics during acute inflammation. (A) Total leukocyte counts were determined by light microscopy, while polymorphonuclear neutrophils (PMNs), classical Ly6C^{hi} monocytes, F4/80^{+} macrophages (MΦs), non-classical monocytes, and the efferocytosis of apoptotic cells were assessed by flow cytometry. (B-D) Lipid mediator profiling of peritoneal lavages from WT mice treated with either an anti-PLXC1 antibody or an IgG control antibody was performed via LC-MS/MS analysis at 4 hours post ZyA treatment. The profiles included mediators derived from arachidonic acid (AA) (B), eicosapentaenoic acid (EPA) (C), and docosahexaenoic acid (DHA) (D). Significance was determined using the unpaired two-tailed Student's t-test: *P < 0.05; **P < 0.01; ***P < 0.001; ****P < 0.0001.

Suppl. Figure 4. Validation of PLXC1 expression in peritoneal cells from PLXC1^{+}/$^{+}$ and PLXC1^{-}/$^{-}$ mice. Flow cytometric analysis of PLXC1 expression in peritoneal cells isolated from PLXC1^{+}/$^{+}$ and PLXC1^{-}/$^{-}$ mice. Results are displayed as box-and-whisker plots (minimum to maximum, all individual data points displayed; n = 4–5 per group). Statistical analysis was performed using an unpaired two-tailed t-test.

Supplementary Figure 5. Representative Western blot analysis of macrophage polarization and PLXC1 expression. (A) Protein expression of CD80 and (B) CD40 in human monocyte-derived macrophages following stimulation with IL-1β (1 ng/ml), IL-1β + anti-PLXC1 antibody (1 μg/ml), or IL-1β + IgG isotype control (1 μg/ml). (C) Expression of PLXC1 in GM-CSF– and M-CSF–differentiated macrophages under control conditions and after stimulation with IL-1β. GAPDH or γ-Tubulin served as a loading control. Representative blots from 2-3 independent experiments are shown.

Supplementary Figure 6. Phagocytosis and efferocytosis assays illustrating Plexin C1-dependent effects. (A) Representative fluorescence microscopy images of zymosan A (ZyA) particle uptake in human macrophages treated with IgG isotype control or increasing concentrations of anti-PLXC1 antibody (0.5, 1, and 2 µg/ml). Images show staining for DAPI (nuclei, blue), FITC-labeled ZyA particles (green), and rhodamine-phalloidin (F-actin, red); merged overlay images demonstrate internalized particles. Scale bar = 10 µm. (B) Quantification of phagocytosis of apoptotic PMNs by human macrophages following co-stimulation with LPS (100 ng/ml). Data are presented as box-and-whisker plots (minimum to maximum, all individual values shown) from three independent technical replicates. (C) Representative fluorescence microscopy images illustrating the uptake of apoptotic PMNs under the indicated conditions. Statistical analysis was performed using one-way ANOVA followed by Dunnett's multiple-comparison test.

Suppl. Figure 7. Expression of alternative "find-me" signal receptors following PLXC1 blockade. Quantitative real-time PCR analysis of receptor expression in human monocyte-derived macrophages after stimulation with IL-1β, IL-1β + anti-PLXC1 antibody, or IL-1β + IgG isotype control. Human PBMCs were stimulated with GM-CSF in RPMI 1640 medium supplemented with 10% fetal calf serum (FCS) for 7 days to differentiate into M1 macrophages. (A) CD88, (B) S1PR1, and (C) G2A mRNA expression levels are shown as box-and-whisker plots (minimum to maximum, all individual data points displayed; n = 6–12 per group). Data were normalized to 18S and expressed relative to IL-1β control conditions. Statistical analysis was performed using one-way ANOVA followed by Dunnett's multiple-comparison test.

Suppl. Figure 8. TNF-α levels in peritoneal lavage exudates following PLXC1 antibody treatment. ELISA quantification of TNF-α concentrations in peritoneal lavage fluid collected 4 hours after zymosan A–induced peritonitis from mice treated with IgG isotype control or anti-PLXC1 antibody (1 µg/ml). Data are presented as box-and-whisker plots (minimum to

maximum, all individual values shown; n = 5–7 per group). Statistical analysis was performed using an unpaired two-tailed t-test.
