# Supplementary figures and images for: Plexin C1 modulates metabolic programming for resolution of severe inflammation
Source: Cell Commun Signal. 2025 Nov 25;23:523. doi: 10.1186/s12964-025-02518-z (PMC12690942; doi:10.1186/s12964-025-02518-z)

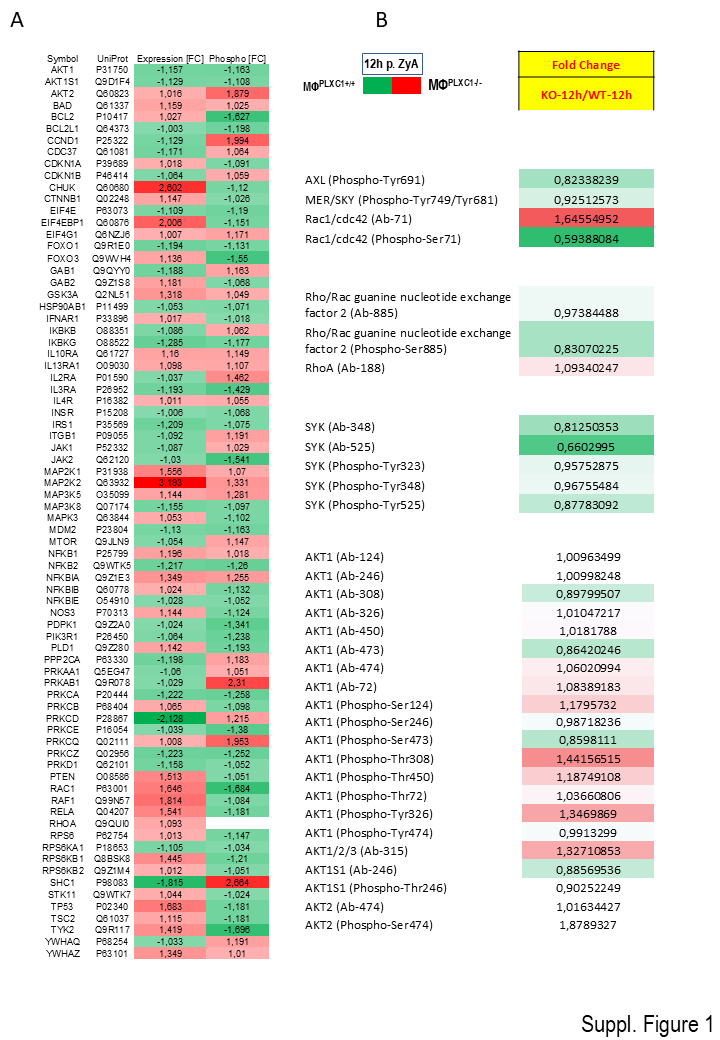

Supplement: Supplementary file 2 — Supplementary Material 2; Supplementary Figure 1. [file 12964_2025_2518_MOESM2_ESM.tif]

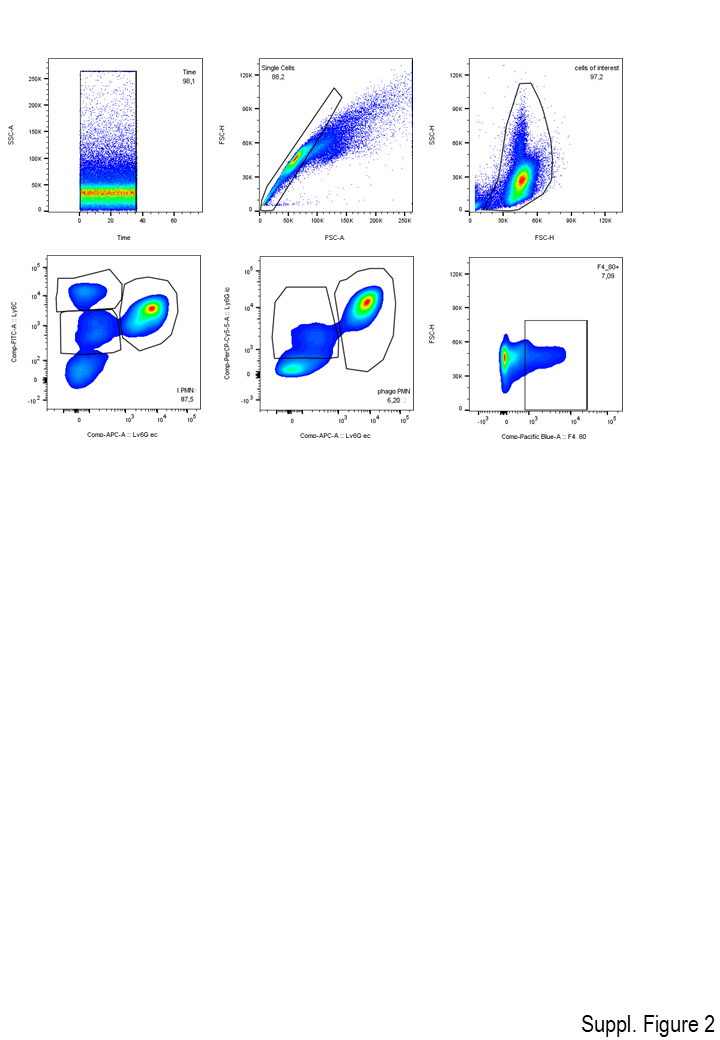

Supplement: Supplementary file 3 — Supplementary Material 3; Supplementary Figure 2. [file 12964_2025_2518_MOESM3_ESM.tif]

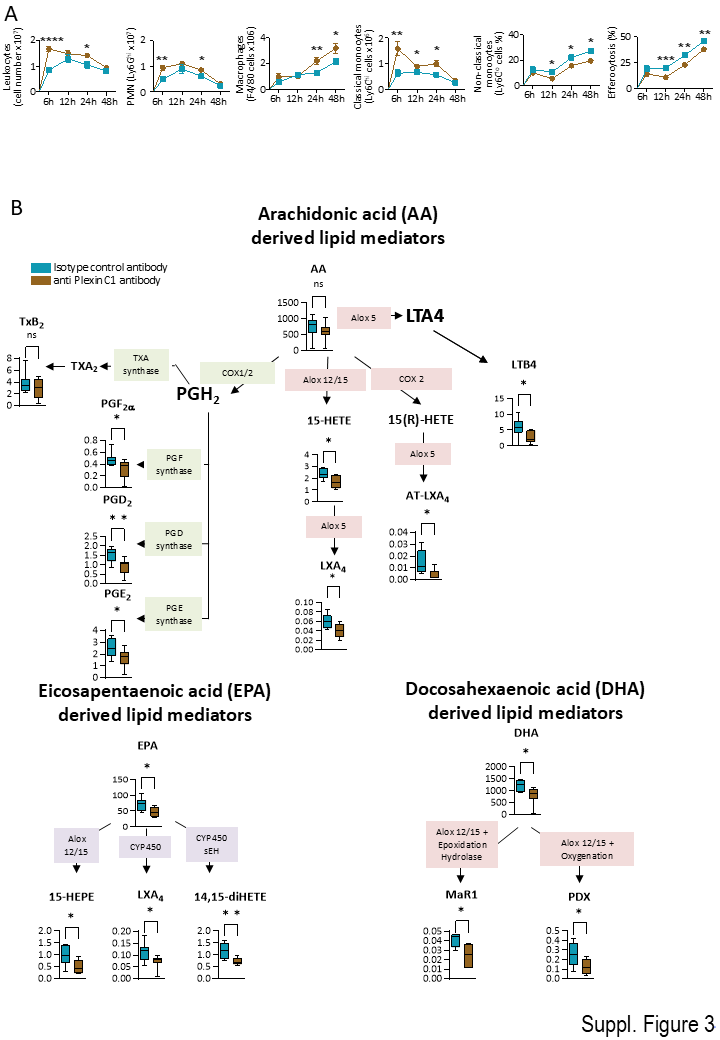

Supplement: Supplementary file 4 — Supplementary Material 4; Supplementary Figure 3. [file 12964_2025_2518_MOESM4_ESM.tif]

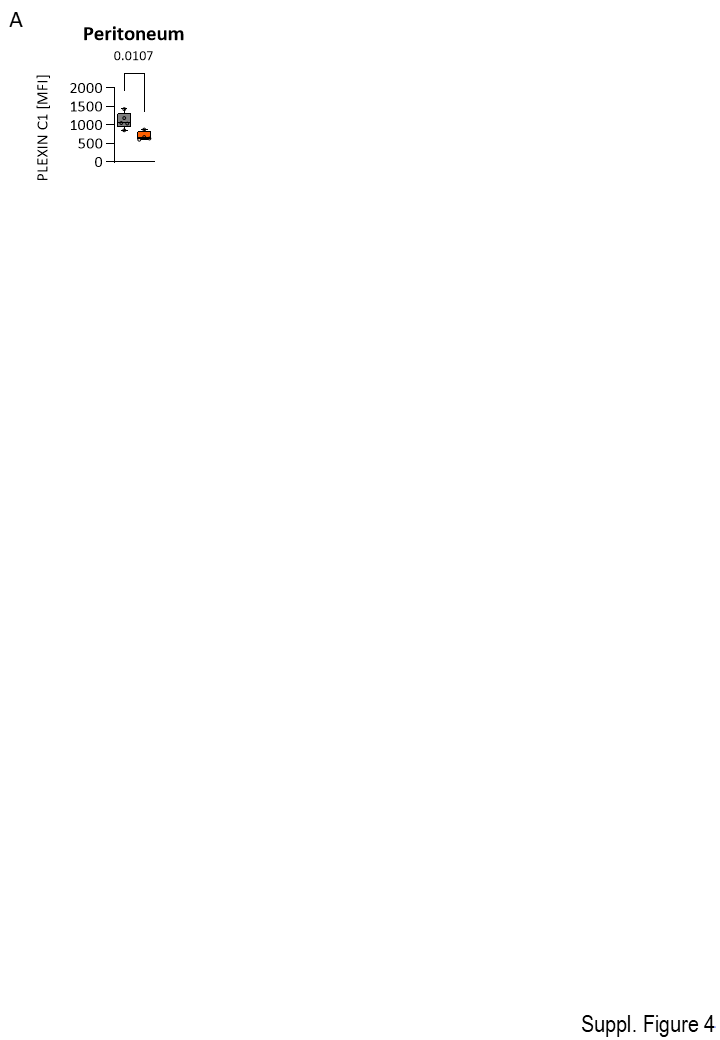

Supplement: Supplementary file 5 — Supplementary Material 5; Supplementary Figure 4. [file 12964_2025_2518_MOESM5_ESM.tif]

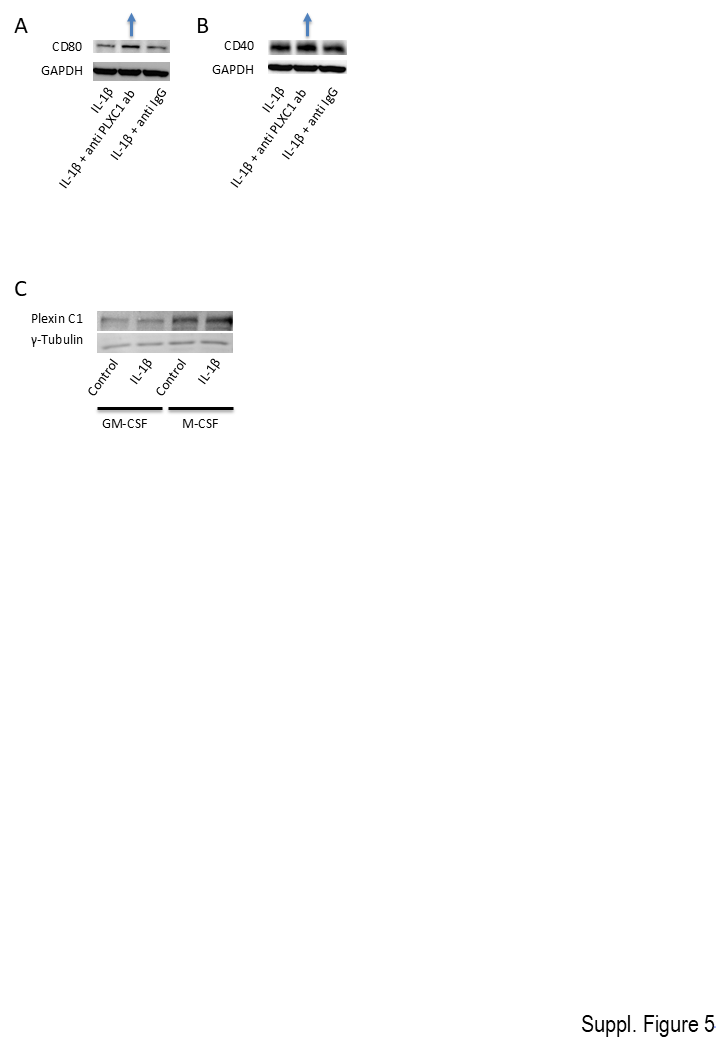

Supplement: Supplementary file 6 — Supplementary Material 6; Supplementary Figure 5. [file 12964_2025_2518_MOESM6_ESM.tif]

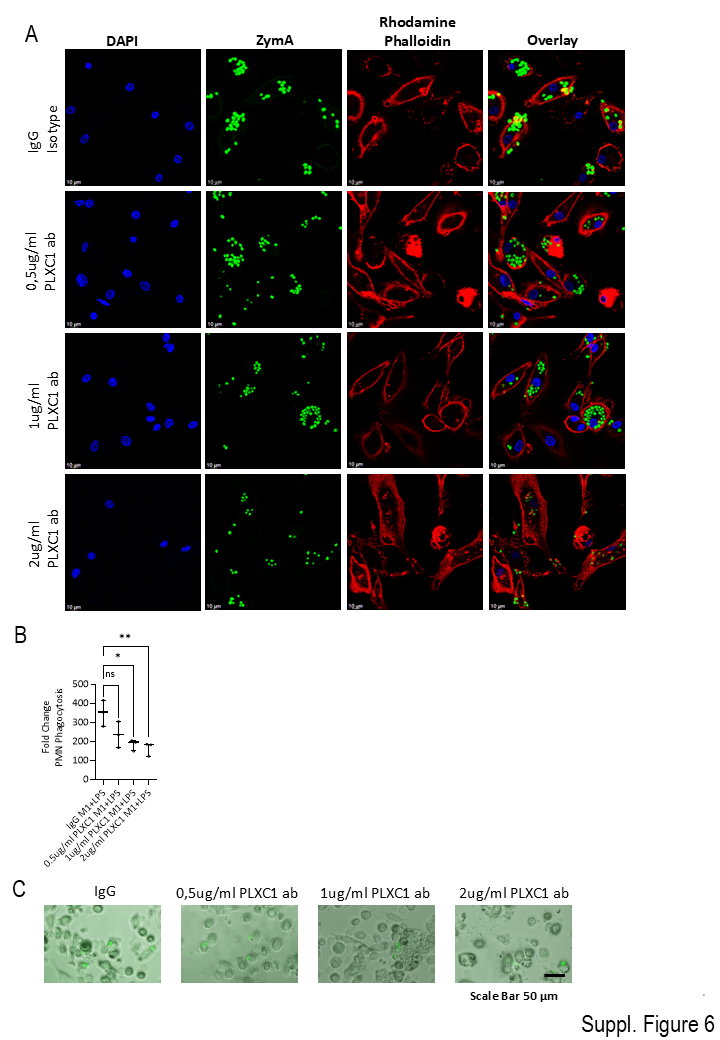

Supplement: Supplementary file 7 — Supplementary Material 7; Supplementary Figure 6. [file 12964_2025_2518_MOESM7_ESM.tif]

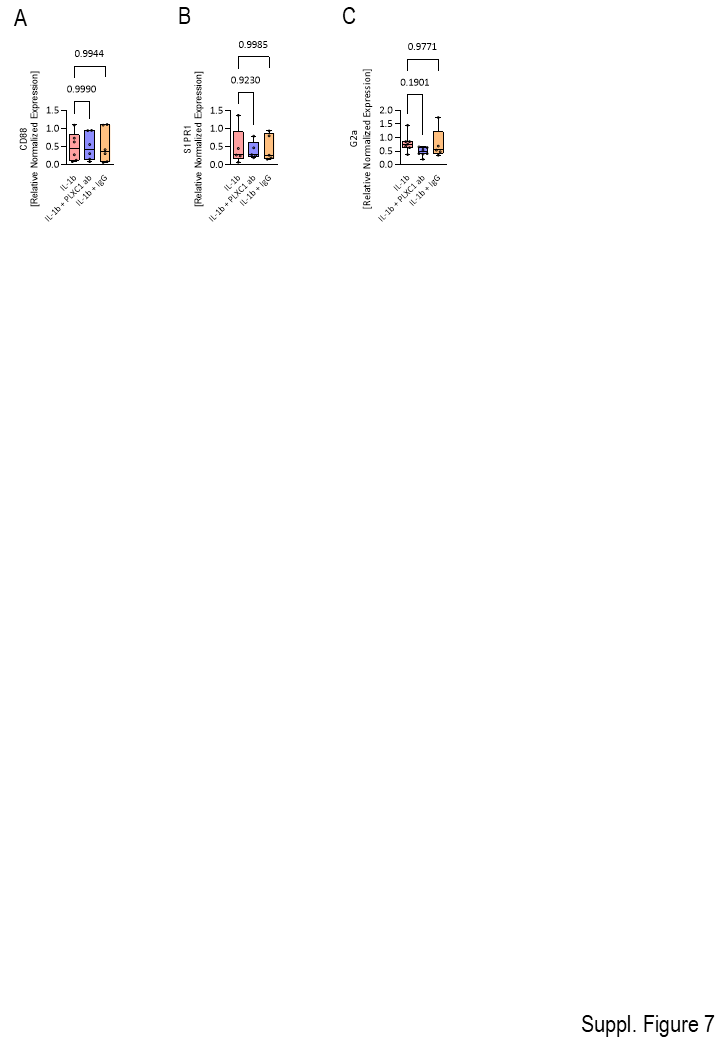

Supplement: Supplementary file 8 — Supplementary Material 8; Supplementary Figure 7. [file 12964_2025_2518_MOESM8_ESM.tif]

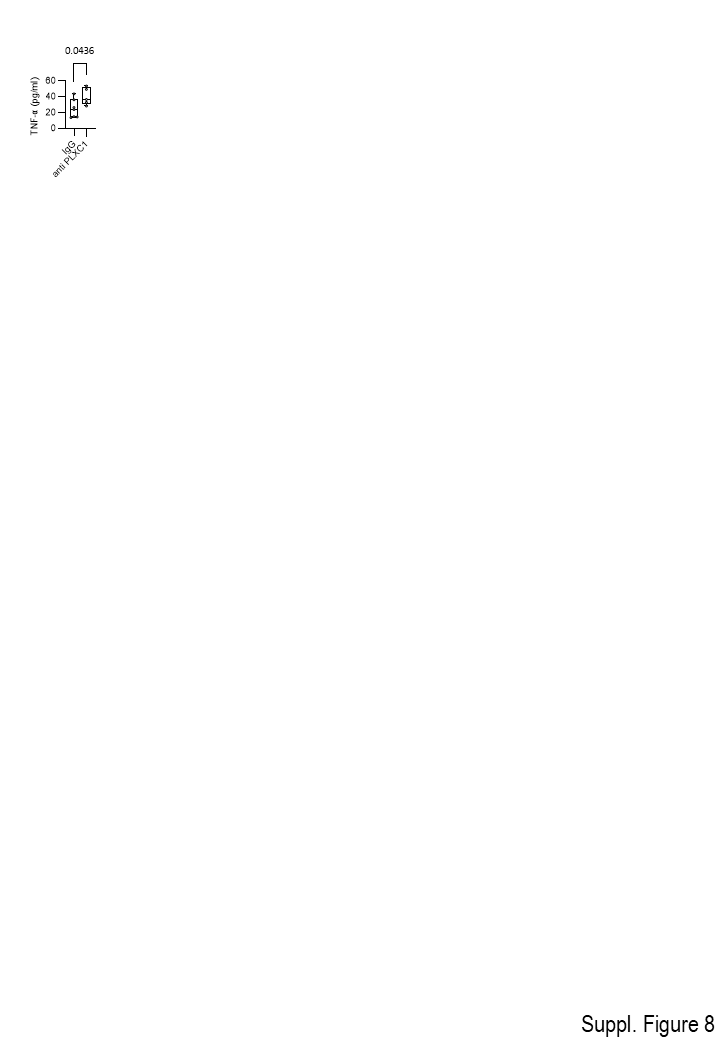

Supplement: Supplementary file 9 — Supplementary Material 9; Supplementary Figure 8. [file 12964_2025_2518_MOESM9_ESM.tif]

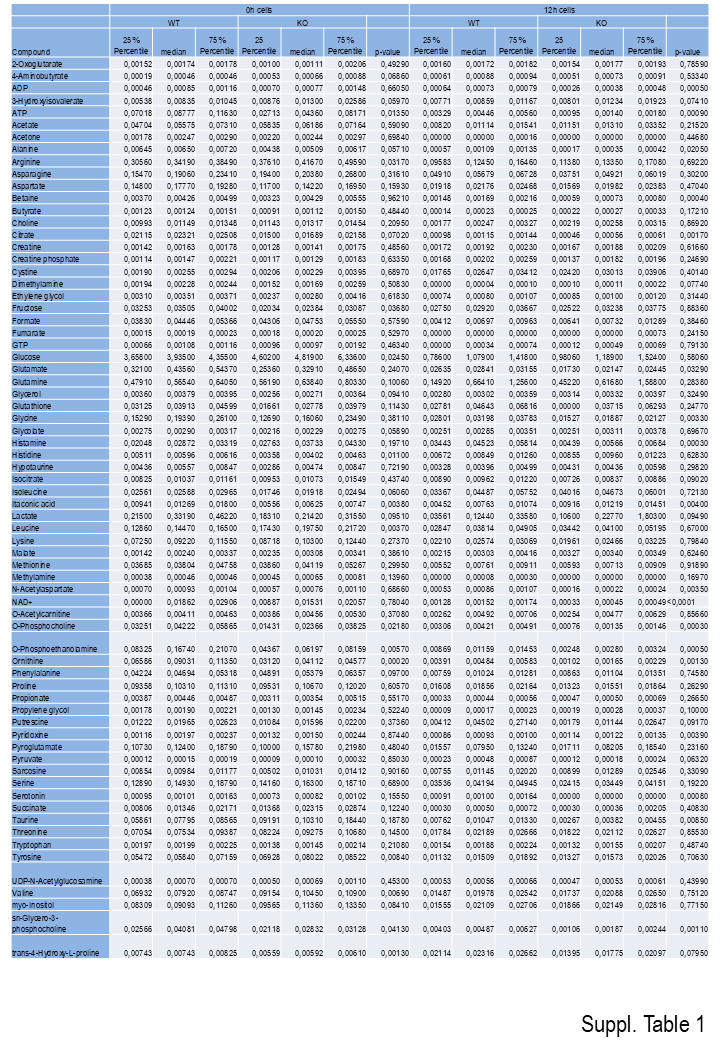

Supplement: Supplementary file 10 — Supplementary Material 10; Supplementary Table 1. [file 12964_2025_2518_MOESM10_ESM.tif]

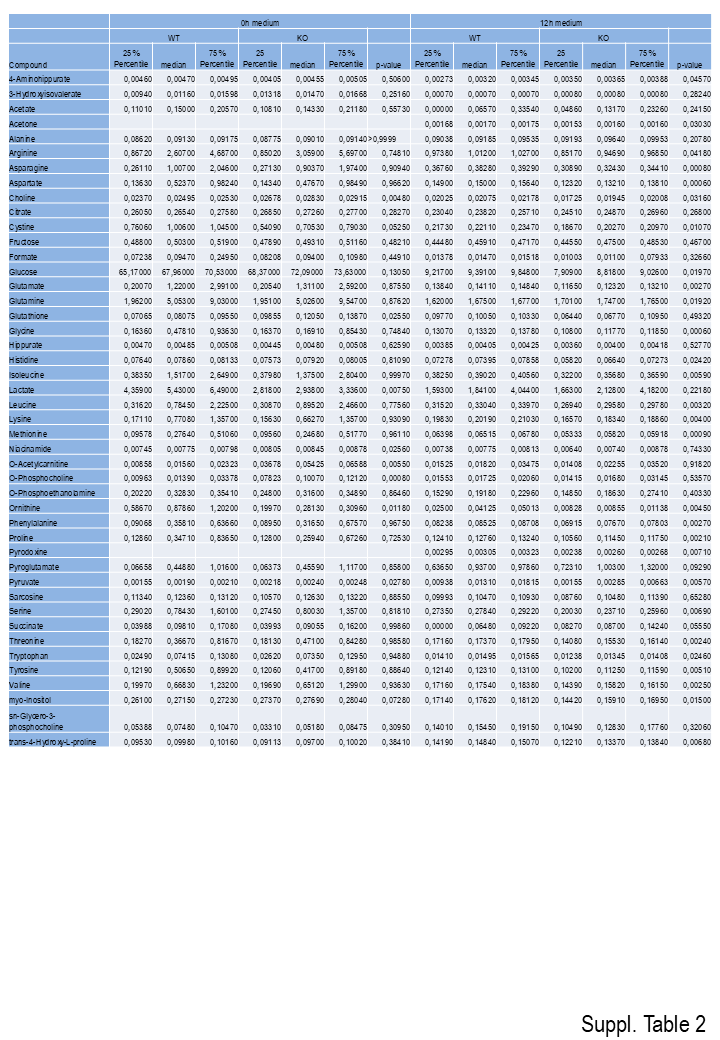

Supplement: Supplementary file 11 — Supplementary Material 11; Supplementary Table 2. [file 12964_2025_2518_MOESM11_ESM.tif]

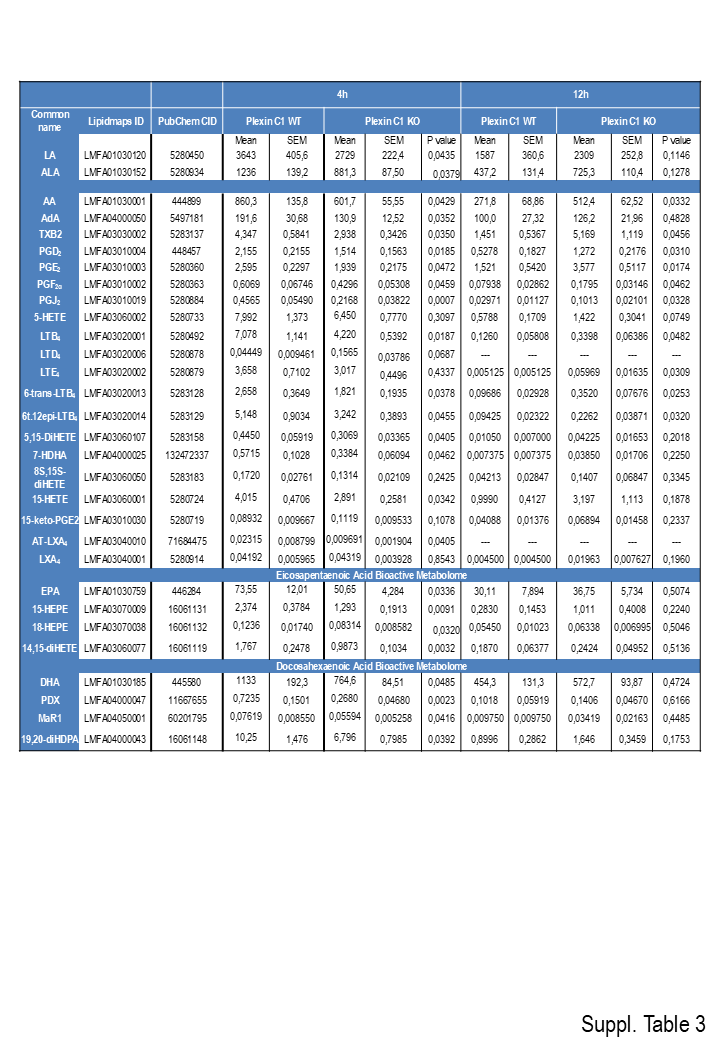

Supplement: Supplementary file 12 — Supplementary Material 12; Supplementary Table 3. [file 12964_2025_2518_MOESM12_ESM.tif]

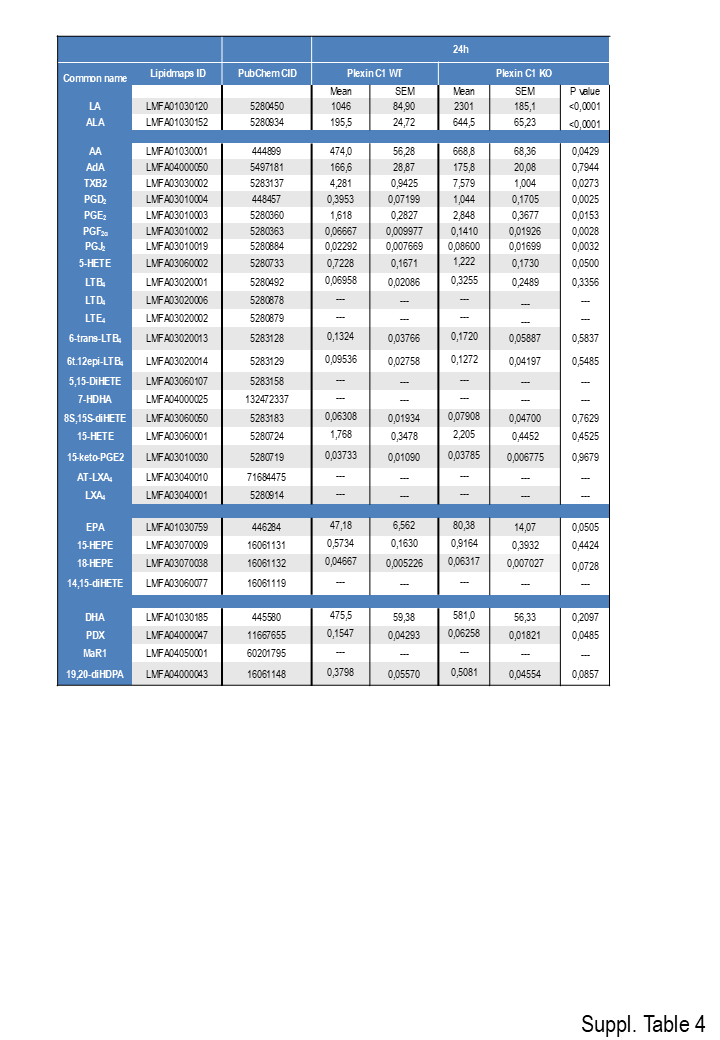

Supplement: Supplementary file 13 — Supplementary Material 13; Supplementary Table 4. [file 12964_2025_2518_MOESM13_ESM.tif]
